# Functionalization of Indium Oxide for Empowered Detection of CO_2_ over an Extra-Wide Range of Concentrations

**DOI:** 10.1021/acsami.3c04789

**Published:** 2023-06-30

**Authors:** A. Rossi, B. Fabbri, E. Spagnoli, A. Gaiardo, M. Valt, M. Ferroni, M. Ardit, S. Krik, A. Pedrielli, L. Vanzetti, V. Guidi

**Affiliations:** † Department of Physics and Earth Sciences, University of Ferrara, Via Saragat 1, Ferrara 44122, Italy; ‡ MNF- Micro Nano Facility, Sensors and Devices Center, Bruno Kessler Foundation, Via Sommarive 18, Trento 38123, Italy; § Institute for Microelectronics and Microsystems IMM-CNR, via Gobetti 101, 40129 Bologna, Italy; ∥ Department of Civil, Environmental, Architectural Engineering and Mathematics (DICATAM), Università degli Studi di Brescia, Via Branze, 43, 25123 Brescia, Italy; ⊥ Sensing Technologies Lab, Faculty of Engineering, Free University of Bozen-Bolzano, Piazza Università 5, Bolzano 39100, Italy

**Keywords:** CO_2_ detection, chemically active metal oxides, In_2_O_3_, operando spectroscopies, smart sensors for IoT

## Abstract

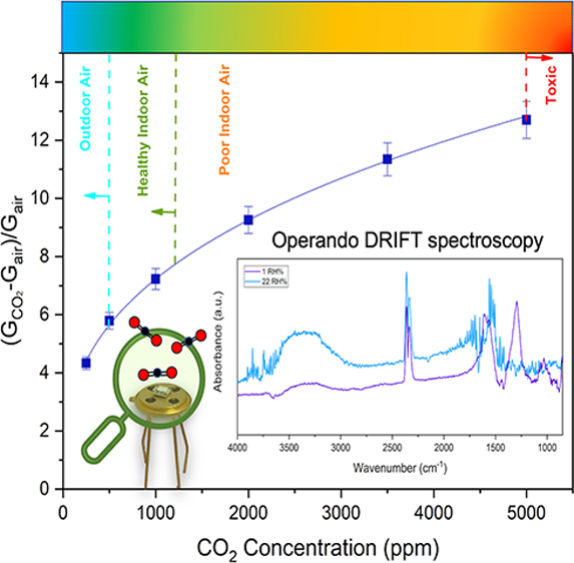

Carbon capture, storage, and utilization have become familiar terms when discussing climate change mitigation actions. Such endeavors demand the availability of smart and inexpensive devices for CO_2_ monitoring. To date, CO_2_ detection relies on optical properties and there is a lack of devices based on solid-state gas sensors, which can be miniaturized and easily made compatible with Internet of Things platforms. With this purpose, we present an innovative semiconductor as a functional material for CO_2_ detection. A nanostructured In_2_O_3_ film, functionalized by Na, proves to enhance the surface reactivity of pristine oxide and promote the chemisorption of even rather an inert molecule as CO_2_. An advanced *operando* equipment based on surface-sensitive diffuse infrared Fourier transform is used to investigate its improved surface reactivity. The role of sodium is to increase the concentration of active sites such as oxygen vacancies and, in turn, to strengthen CO_2_ adsorption and reaction at the surface. It results in a change in film conductivity, i.e., in transduction of a concentration of CO_2_. The films exhibit excellent sensitivity and selectivity to CO_2_ over an extra-wide range of concentrations (250–5000 ppm), which covers most indoor and outdoor applications due to the marginal influence by environmental humidity.

## Introduction

In recent years, with the development of industry and exponential urbanization, the problems of air pollution and global warming have become increasingly serious and gas monitoring has attained a sufficiently wide market to be commercially attractive. Carbon dioxide (CO_2_) is considered as the most important contributor to global warming, accounting for 76% of the greenhouse effect,^1,2^ with an average global concentration level of 420 parts per million (ppm).^3^ In addition, recent research highlighted that people spend more than 90% of their time in indoor environments^4^ and, consequently, are subjected to breathe CO_2_ concentrations higher than the environmental background up to several thousand ppm, depending on the occupancy and ventilation of the confined space.^5^ Indeed, high concentrations of indoor CO_2_ have been linked to a deleterious health effect, such as the sick building syndrome.^6^ In particular, long exposure to concentrations ranging between 2000 and 5000 ppm negatively affects cognitive performance, including headaches and loss of attention.^7^ Severe toxicity and oxygen deprivation effects occur as the CO_2_ concentration exceeds 5000 ppm over an 8 h workday.^8^

Moreover, as recently reported by Peng and Jimenez (2021), CO_2_ was pointed out as an indicator for indoor ventilation and in turn of pathogen infection (e.g., SARS-CoV-2) probability through aerosol transmission.^9^ Monitoring CO_2_ concentration could be useful in greenhouse planting (where the CO_2_ concentration is usually kept below 300 ppm) and in packaging for conservation of fruits and vegetables (up to 25%).^10,11^

For CO_2_ measurements, several techniques have been developed, spanning from analytical instruments such as gas chromatography (GC) assisted by mass spectrometry (MS), infrared spectroscopy (IR) to compact and portable devices, i.e., optical, acoustic, electrochemical, capacitive, and nondispersive infrared-based sensors (NDIR). However, some limitations, e.g., lack of portability, high-maintenance cost, need for trained users (for GC–MS), short device lifetime (for optical and electrochemical sensors), low selectivity (for acoustic sensors), and spectral interference (for NDIR sensors), have prevented CO_2_ monitoring on a large scale.^12−15^

Hence, alternative systems combining accuracy, resolution, and robustness with small size, low cost, and power consumption would be highly demanded. Solid-state gas sensors, such as chemoresistive devices, would represent a viable route in this sense to previously mentioned tools, because such devices could be easily integrated in Internet of Things (IoT) networks. Among them, metal-oxide (MOX)-based gas sensors have gained a wide market due to high sensitivity, rapid response, stability, and reproducibility combining simple and low-cost fabrication methods. Unfortunately, to date, they have exhibited a modest attitude to detect CO_2_, resulting in a poor response due to the inherent stable nature of such a molecule.^16−21^

Therefore, new functional materials for CO_2_ sensing featuring stronger chemical reactivity, while maintaining complete reversibility of the detection process, represent a challenge. MOXs are an interesting class of semiconductors, as their properties can be engineered by changing the chemical–physical composition of the MOX, i.e., the crystal structure, the size and shape at the nanostructure level, or by introducing additives (main-group elements, transition metals, noble metals, etc.).^22,23^ In particular, dopant MOXs proved effective to enhance chemical reactivity.^24^ Recently, it was discovered that alkali metals act as catalysts to promote analyte adsorption at the compound surface.^25^ In fact, they accelerate the formation and facilitate the stabilization of reaction intermediates, ultimately increasing the adsorption of CO_2_.

Indium oxide (In_2_O_3_) has been studied in electrochemistry as an efficient catalyst for CO_2_ hydrogenation to methanol.^26^ The basic idea behind this investigation is to merge the concepts above, i.e., develop a sodium-doped indium oxide semiconductor (Na:In_2_O_3_) to achieve high CO_2_ sensing. Indeed, In_2_O_3_ is not a novelty among the MOX sensors, because its detection properties have already been probed vs ozone, nitrogen dioxide, and methane.^27−29^ In sight of potential large-scale applications, we resorted to a simple synthesis method such as the sol–gel process. The powders, conveniently characterized, were used as a functional component for screen-printed sensing films and probed vs different concentrations of CO_2_ and its potential interfering gases (toluene, ethanol, carbon monoxide, and nitrogen dioxide) for applications. The sensing capability vs CO_2_ was explored over a wide range of concentrations (250–5000 ppm), covering most relevant indoor and outdoor applications. The role of the sodium in the sensing mechanism was investigated through Fourier transform infrared diffuse reflectance (DRIFT) spectroscopy, an *operando* characterization technique to monitor the gas–solid interaction occurring at the surface while the sensor is working.^30−32^

## Experimental Section

In this study, we synthesized pristine and Na-doped indium oxide by means of the sol–gel process. The morphology, elemental composition, and structure of the nanopowders were investigated by scanning electron microscopy (SEM), transmission electron microscopy (TEM), electron X-ray diffraction (EDX) analysis, and X-ray diffraction (XRD). The doping effect of sodium incorporation in the indium oxide nanostructure was derived from X-ray photoelectron spectroscopy (XPS) and optical properties obtained by UV–visible (UV–vis) measurements. Then, sensing devices were produced by a scalable and controllable fabrication technique such as screen printing. The sensors based on pristine and Na-doped indium oxide were electrically characterized to study their sensing performance. Finally, the impact of sodium addition on the latter was investigated through *operando* DRIFT spectroscopy.

### Materials

Indium(III) nitrate hydrate (99.9%) (In(NO_3_)_3_·5H_2_O) was purchased from Sigma-Aldrich, USA. Sodium hydroxide anhydrous pellets (NaOH), ammonia (NH_3_), and propan-2-ol (C_3_H_8_O) were purchased from CARLO ERBA Reagents S.A.S. Deionized (DI) water was procured from the Millipore DI water purification system.

### Synthesis and Film Deposition

#### Synthesis of In_2_O_3_ Powder

In_2_O_3_ nanopowder was prepared by the sol–gel method. In(NO_3_)_3_·5H_2_O (0.1 M) was dissolved in 60 mL of DI water. Then, 4.0 mL of NH_3_ was added to the previously prepared aqueous solution. The mixture was stirred for 30–40 min at room temperature.

#### Synthesis of Na:In_2_O_3_ Powder

Na:In_2_O_3_ powder was synthetized through the sol–gel technique. First, 0.1 M In(NO_3_)_3_·5H_2_O was dissolved in 60 mL of DI water. Then, 0.5 M NaOH was added to the above precursor aqueous solution and stirred for 40 min at 70 °C.

The slurry, obtained from the two syntheses, was washed with isopropanol and DI water several times using a centrifuge at 5000 rpm for 2 min. The white precipitate was dried at 100 °C for 4 h and consecutively at 200 °C for 2 h. The dried powder was thermally treated at 450 °C for 3 h in an ambient air.

Details on material characterization methods (XRD, SEM, TEM, XPS, and UV–vis) are reported in the Supporting Information.

#### Film Deposition

The yellow powders were ground in an agate mortar and mixed with α-terpineol, ethyl cellulose, and silica to form a homogeneous paste (step 1 in Figure S1, Supporting Information).

The resulting composites were screen printed by an AUREL C920 onto alumina substrates, commercially available, owing two interdigitated gold electrodes on the front-side, and a platinum heater on the backside to thermo-activate the sensing layer of the device (step 2; Figure S1). In particular, the electrodes supply the input voltage (5 V) and extract the output signal of the sensing layer. The printed film (∼20 μm thick) was calcined at 450 °C for 3 h in air and finally packaged by welding, with a thermo-compression wedge wire bonder, the four contacts to a TO-39 support, commercially available, using gold wires with a diameter of 0.06 mm^33^ (step 3; Figure S1).

### Gas Sensing Measurements

The electrical characterization was developed through two approaches to deeply investigate the gas sensing performance of In_2_O_3_-based sensors:

- *Electrical characterization*: experimentation in a standard test chamber for identifying the optimal working conditions, sensitivity, repeatability, response and recovery times, humidity influence, and selectivity.
- *Sensing mechanism investigation*: *operando* DRIFT investigation of the Na:In_2_O_3_ sensor exposed to CO_2_ in dry and wet conditions.

### Electrical Characterization Setup

The sensing properties of the Na:In_2_O_3_ film were tested in a 622 cm^3^ gas-flow sealed chamber. Synthetic air (20% O_2_ and 80% N_2_) and target gases from certified cylinders (N5.0 degree of purity) were mixed and fluxed through mass-flow controllers at 500 standard cc/min (sccm). Then, the filling time of the test chamber was calculated to be about 1 min and 15 s, as it depends on the size, the geometry of the chamber, and the velocity of the gas flow.^34^

Relative humidity and temperature inside the test chamber were controlled by a commercial Honeywell HIH-4000 humidity sensor. The test chamber was placed in a climatic box, which maintains a constant outer temperature of 25 °C through a thermal ventilation system.^35^

### Electrical Measurements

#### Working Temperature

Sensors were kept at their optimal working temperature, identified after a proper calibration with temperatures ranging from 150 to 300 °C, under a continuous flow of synthetic air, until the thermodynamic steady state was attained. The sensor response was defined as
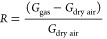
1where *G*_gas_ and *G*_dry air_ are the steady-state conductance in gas and in air, respectively. The response (τ_res_) and recovery (τ_rec_) times were calculated as the time needed to reach 90% of the steady response and the time to restore 90% of the baseline level, respectively.

#### Sensitivity

The film was exposed to increasing concentrations ranging from 250 to 5000 ppm of CO_2_ to sound out different application scenarios.

#### Repeatability

The Na:In_2_O_3_ film was exposed to four cycles of 400 and 1200 ppm of CO_2_.

#### Humidity Influences

The sensors were stabilized at the beginning of each measurement by keeping the sensors at their working temperature under a continuous flow (500 sccm) of wet air, in the range of 1–75 RH%, by fluxing part of the total flux through a bubbler filled with distilled water. After the stabilization, the sensors were exposed to the target gas.
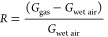
2where *G*_wet air_ is the conductance in wet conditions.

#### Selectivity

The concentrations of selected gases (ethanol, toluene, NO_2_, and CO) were chosen according to the National Institute for Occupational Safety and Health (NIOSH), the American Society of Heating, Refrigerating and Air-Conditioning Engineers (ASHRAE), and the average tested levels reported in the literature.^8,36−39^ The selectivity coefficient (*k*_s_) was defined as the ratio of the response value of the sensors to CO_2_ and interfering gas, respectively.^40^
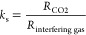
3

#### Limit of Detection (LOD)

According to the International Union of Pure and Applied Chemistry (IUPAC) definition,^41^ the LOD is calculated as LOD = 3(RMS noise/*S*), where RMS noise is the root-mean-square deviation and *S* is the slope of the fitting of the calibration curve.

### *Operando* DRIFT Setup

The kinetics at the gas–solid surface was investigated through *operando* experiments employing a Bruker Vertex 70 V vacuum FTIR spectrometer, equipped with a DRIFT accessory (Praying Mantis, Harrick Scientific Products Inc.) (Figure S2, Supporting Information). The characterization of In_2_O_3_-based sensors was investigated using a dedicated apparatus, including a customized sealed gas test chamber (IR dome with a void volume of ≈0.5 cm^3^) and a data acquisition system. All specifications about the system for gas injection, chamber characteristics, and electronics are given in the Supporting Information and in the previous works.^30,42^

### *Operando* DRIFT Measurements

The single-channel spectrum is composed by the absorption caused by functional groups of species adsorbed on the surface and by the sensing material itself, which together with the diverse individual component of the spectrometer (optical elements, light source emission, and detector nonlinearity) affect the overall shape of the single-channel DRIFT spectrum.

The spectra were acquired through a liquid nitrogen-cooled mercury cadmium tellurium mid-band detector, with a spectral range from 850 to 4000 cm^–1^. The absorbance spectra were calculated by using the equation
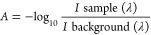
4where *I* sample (λ) and *I* background (λ) are the spectra of the sample and the background.^30,31^

#### Characterization in Dry and Wet Conditions

The samples were maintained at its proper working temperature (200 °C) overnight, and then each spectrum was collected at a resolution of 4 cm^–1^ averaging 1024 scans with a beam spot size of 2.5 mm. All the optical bench settings and spectral data acquisition were performed through Bruker OPUS software.

Before the CO_2_ exposure, the measuring chamber was kept in a constant flow of 100 sccm of synthetic dry air, while the sensor operating temperature was gradually increased with steps of 50 °C up to 200 °C. Each temperature step was maintained for as long as to allow thermodynamically stabilization of the surface. The surface reactivity of the sensor was evaluated after maintaining the device at 200 °C for 1 day under a 100 sccm constant flow of synthetic dry air and an applied voltage between the electrodes of 1 V.^42^ According to these conditions, we evaluated the spectral background for each reported measurement. After a stabilization of the baseline for a few hours, the device was exposed to a mixture of 1000 and 3500 ppm of CO_2_ and synthetic air under dry (≈1% RH at 30 °C) and wet conditions (up to 22 RH% at 30 °C).

## Results and Discussion

### Powder Characterization

**Figure 1:**
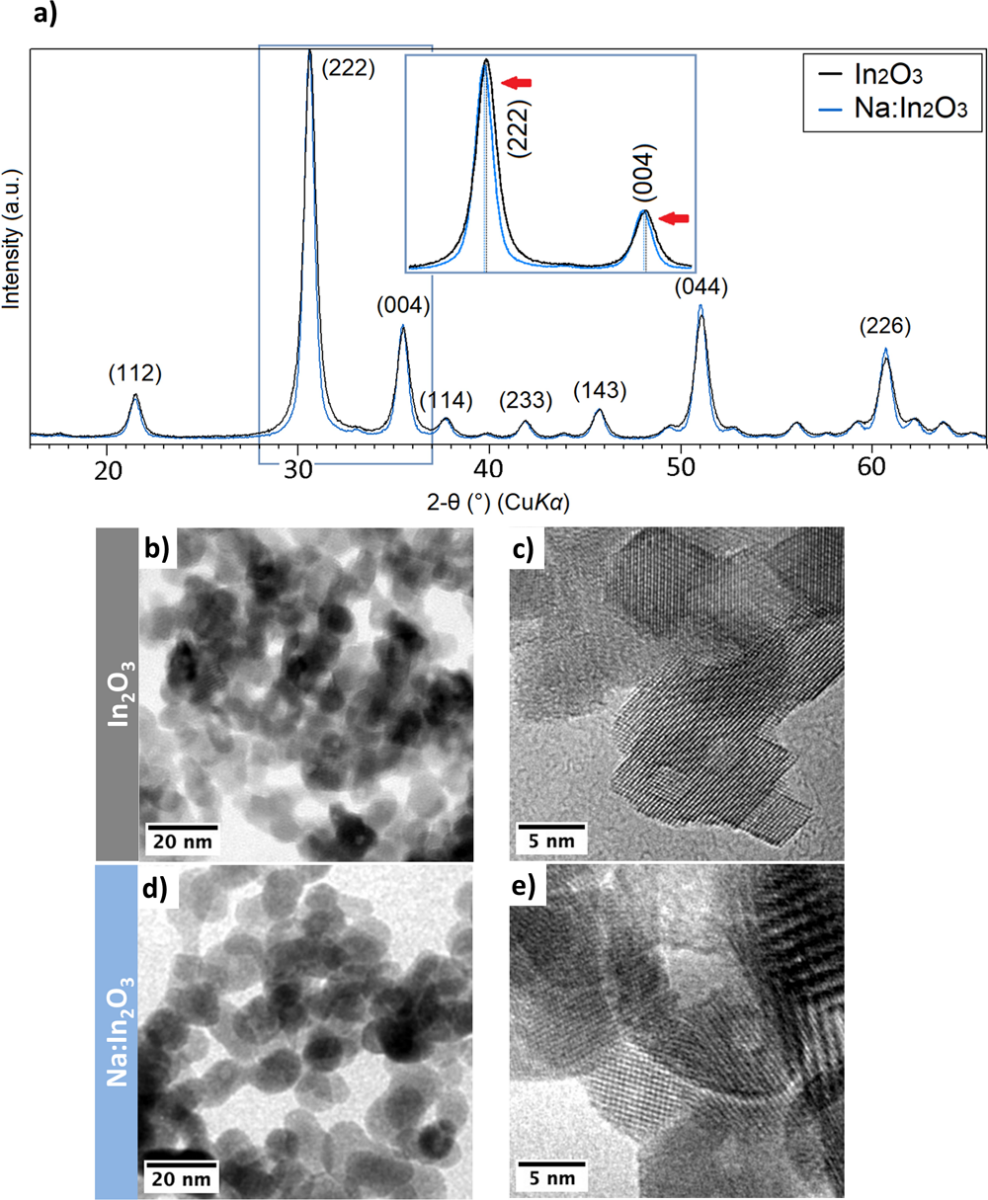
(a) XRD pattern of pristine In_2_O_3_ and Na:In_2_O_3_ powders. Inset: magnification of the Na:In_2_O_3_ peak shift. (b) TEM and (c) HR-TEM images of In_2_O_3_ powder. (d) TEM and (e) HR-TEM images of Na:In_2_O_3_ powder.

The XRD pattern of the same powder revealed that both the pristine and Na-doped samples are monophasic and with a cubic crystal structure (s.g. *Ia*-3) (Figure 1a). The diffraction peaks for Na:In_2_O_3_ were shifted slightly toward 2θ values lower than those for the pristine In_2_O_3_, indicating lattice expansion through sodium doping. Along with a lattice parameter variation, the addition of sodium yielded a crystal structure that has nearly the same crystallite size (*X*_XRD_) but a lower microstrain (*e*_0_), as provided in Table 1. Namely, peak broadening effects that may arise from dislocations or interstitial, substitutional, and other similar point defects as determined by XRD analyses^43^ are hindered by sodium doping.

**Table 1:** Main Crystallographic Information (i.e., Crystal System, Space Group, Lattice Parameter (*a*), Crystallite Size (*X*_XRD_), and Microstrain (*e*_0_)) for the Investigated Samples

sample	crystal system	space group	*a* [Å]	crystallite size, *X*_XRD_ [nm]	microstrain, *e*_0_ × 100
In_2_O_3_	In_2_O_3_ (cubic)	*Ia*-3	10.1193(4)	10.4(1)	0.101(2)
Na:In_2_O_3_	In_2_O_3_ (cubic)	*Ia*-3	10.1213(2)	10.9(1)	0.060(3)

The SEM analysis on the In_2_O_3_-based powders showed regular nanometric spherical particles with an equiaxed shape (Figures S3a and S4, Supporting Information). The morphology of In_2_O_3_ and Na:In_2_O_3_ was further characterized by TEM. Figure 1b,d confirms that the powders consisted of monodisperse nanoparticles and averaged (12 ± 4) nm and (11 ± 3) nm in size for In_2_O_3_ and Na:In_2_O_3_, respectively. Here, crystalline facets were highlighted on the particles with a round shape (see Figure 1c,e). The particles were crystalline as no amorphous or secondary segregated phases were observed at the grain surface. The selected-area electron diffraction pattern (Figure S5, Supporting Information) showed the typical interplanar distances of the cubic phase of In_2_O_3_, the major reflections of which derive from crystalline planes (222), (004), and (044), then confirming XRD results. Furthermore, scanning transmission electron microscopy combined with energy-dispersive X-ray (STEM-EDX) analysis confirmed the localization of Na within the nanometric In_2_O_3_ particles (Figure S6, Supporting Information).

**Figure 2:**
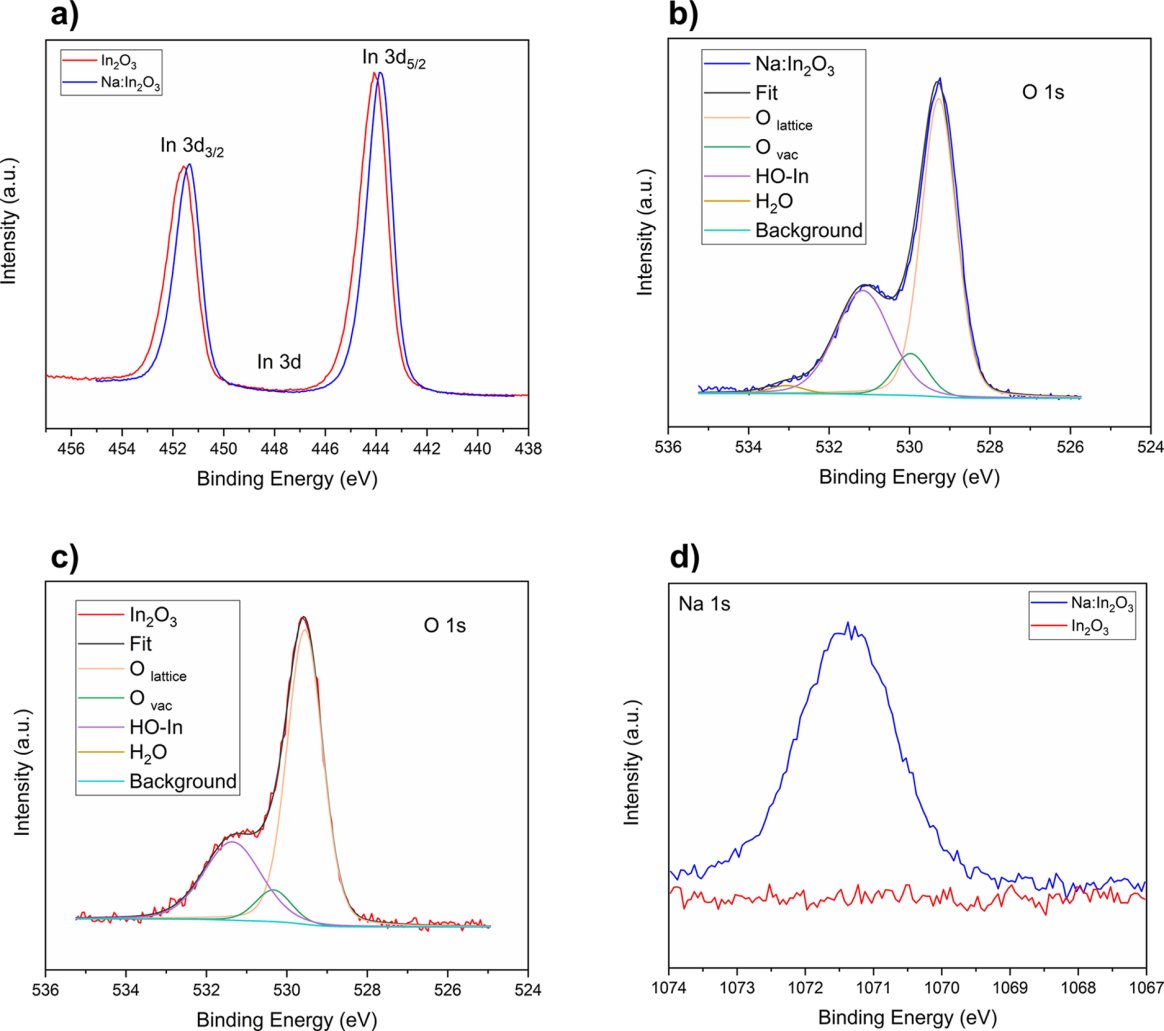
High-resolution XPS spectra of (a) In 3d, (b, c) O 1s, and (d) Na 1s core levels of Na:In_2_O_3_ (blue line) and In_2_O_3_ powders (red line).

The survey spectrum of high-resolution XPS over the powders (Figure 2) revealed the presence of three main elements, i.e., In, O, and Na. To investigate the chemical state of these elements, the high-resolution spectra of In 3d (440–455 eV), O 1s (526–535 eV), and Na 1s (1066–1075 eV) core levels were collected. Their quantification (atomic%) in the pristine and doped samples is reported in Table S1. Figure 2a compares the XPS spectrum of the In 3d core level for the powders with and without Na, which neatly shows the doublet corresponding to 3d_5/2_ and 3d_3/2_. For the pristine In_2_O_3_ sample, In 3d_5/2_ and In 3d_3/2_ peaks appeared at 444.1 and 451.6 eV, respectively. The energy of the In 3d doublet corresponds to the In^3+^ oxidation state and In-O bonds.^44^ A slight change of binding energies of the In 3d peaks was observed between pristine In_2_O_3_ and Na:In_2_O_3_ samples. The displacement of these peaks indicates different chemical neighborhoods due to incorporation of sodium into the In_2_O_3_ lattice. Then, a similar shift can be also observed for O 1s, as reported in Table S2. Figure 2b,c shows a detailed deconvolution of the O 1s high-resolution spectra, both for Na:In_2_O_3_ and In_2_O_3_ samples, in four peaks corresponding to the O lattice (In-O-In), O atoms adjoined to oxygen deficiency sites (oxygen vacancies), surface hydroxyl groups OH-In, and H_2_O (∼533.0 eV).^45−47^ The main O 1s peak at 529.6 eV for In_2_O_3_ (529.2 eV for Na:In_2_O_3_) corresponds to the O lattice. The binding energies at 530.3 eV for In_2_O_3_ (529.9 eV for Na:In_2_O_3_) are assigned to O atoms adjoined to oxygen vacancies, while the O 1s peak at 531.4 eV for In_2_O_3_ (531.1 eV for Na:In_2_O_3_) is related to the adsorbed −OH terminations.^48,49^ The increased % of O atoms adjoined to oxygen vacancies found in the doped In_2_O_3_ sample suggests a higher concentration of oxygen vacancies for Na:In_2_O_3_ (8.1%) than for In_2_O_3_ (7.0%). Although the quantification of the total amount of oxygen vacancies in indium oxide by XPS analysis can be hardly deduced from the fit of the O 1s peaks, the assessment is supported by the different ratios between oxygen and metals (In + Na) in the two samples, equal to 1.22 and 1.32 for Na:In_2_O_3_ and In_2_O_3_, respectively (Table S1).

Figure 2d shows the high-resolution spectrum of the Na 1s core level in the Na:In_2_O_3_ powder. The binding energy identified at 1071.4 eV is ascribed to Na^+^,^50^ which confirmed the successful incorporation of Na into the In_2_O_3_ lattice through sol–gel synthesis. Therefore, doping with low-oxidation-state alkali metals is a suitable strategy to promote the formation and to control the concentration of oxygen vacancies in MOX, enabling the tuning of catalytic and optoelectronic properties, as already observed in previous works.^51,52^

**Figure 3:**
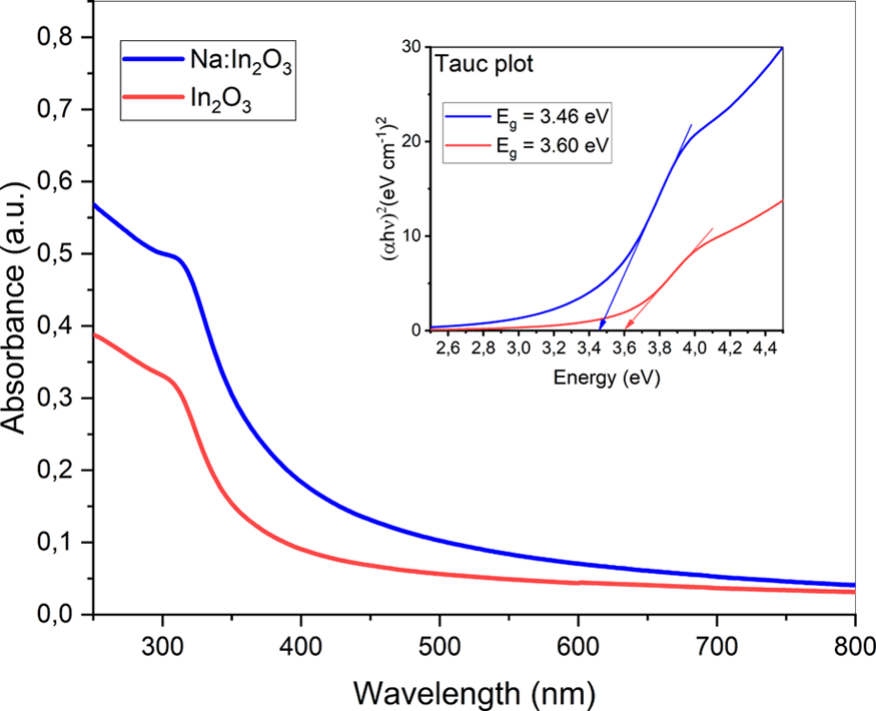
UV–vis absorbance spectrum and inset of (α*h*ν)^2^ vs energy (eV) for Na:In_2_O_3_ and In_2_O_3_.

The optical properties of the In_2_O_3_-based nanopowders were characterized by UV and visible spectroscopies (Figure 3). It can be observed that the maximum in the absorption spectrum for pristine In_2_O_3_ was recorded at a wavelength of about 304 nm. For comparison, the maximum for doped In_2_O_3_ was red-shifted to 313 nm. Specifically, the optical absorption of the powders was investigated to account for the influence of sodium to the direct band gap (*E*_g_) of In_2_O_3_. The latter was determined by using Tauc’s plot,^53^ resulting in *E*_g_ = 3.60 and 3.46 eV for the pristine In_2_O_3_ and Na:In_2_O_3_, respectively. This observation confirmed the role of sodium as a dopant (Na^+^). In fact, if sodium had been present in the sample as metallic (Na^0^), no decrease in the band gap width would have been observed.^54,55^ This decrease in the band gap confirms the role of Na^+^ cations as an electrically active dopant in In_2_O_3_.

### Gas Sensing Performance

The gas response of the films based on pristine In_2_O_3_ and Na:In_2_O_3_ was investigated by measuring the conductance change at different working temperatures (150–450 °C) under exposure to 500 ppm of CO_2_. As shown in Figure S7a,b (Supporting Information), the optimal working temperature for the Na:In_2_O_3_ film lied in the range of 200–250 °C, while the In_2_O_3_ film peaked at 250 °C. In this work, to meet the demand for lowest power consumption, an operating temperature of 200 °C was chosen hereinafter for Na:In_2_O_3_. As expected, the conductance change after the injection of reducing gas such as CO_2_ (Figure S7c) increased for both In_2_O_3_ and Na:In_2_O_3_, owing to their n-type behavior. Then, the sensor responses were calculated using eq 1.^34^

**Figure 4:**
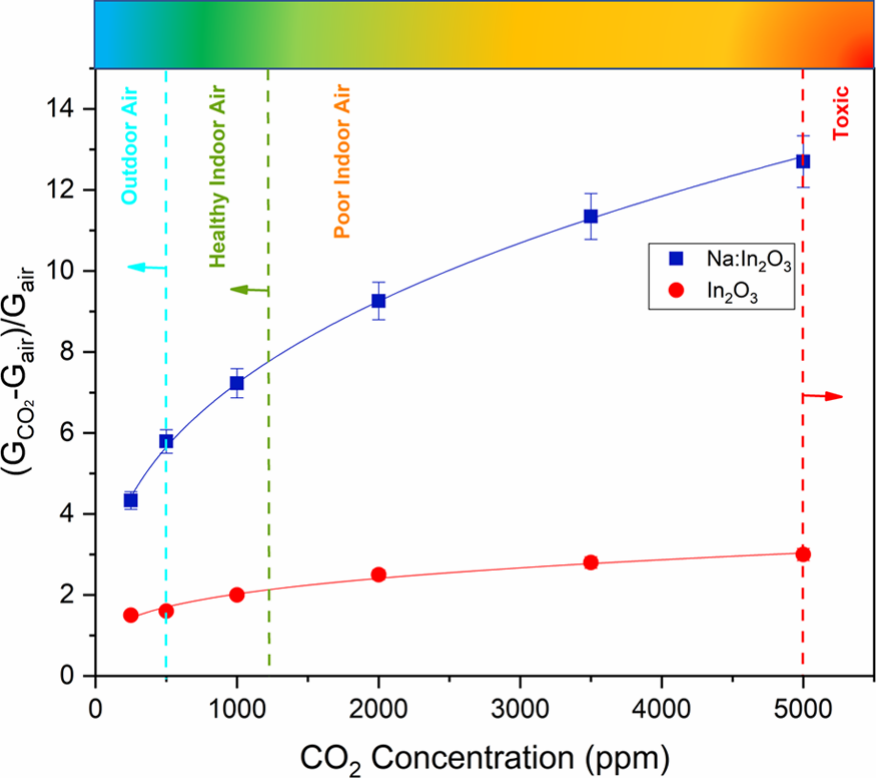
Calibration curves of Na:In_2_O_3_ and pristine In_2_O_3_ sensors at 200 and 250 °C, respectively, for CO_2_. During the measurements, the temperature and humidity inside the chamber were 25 °C and 2 RH%, respectively.

The sensitivity to CO_2_ was investigated by measuring the conductance of the Na:In_2_O_3_ film when exposed to 250, 500, 1000, 2000, 3500, and 5000 ppm of CO_2_, as compared to that for pristine In_2_O_3_. The gas concentration range was selected to probe the sensor for operation in both indoor and outdoor applications.^11,56,57^ As it can be seen in Figure 4, the response of the Na:In_2_O_3_ film gradually increased from 4.33 at 250 ppm to 12.7 at 5000 ppm, featuring remarkable sensitivity that envisages noteworthy potential for CO_2_ monitoring in several contexts. In contrast, the response of the pristine In_2_O_3_ film was much lower than for the doped film and, above all, with strong tendency to saturate even at the lowest concentrations.

**Figure 5:**
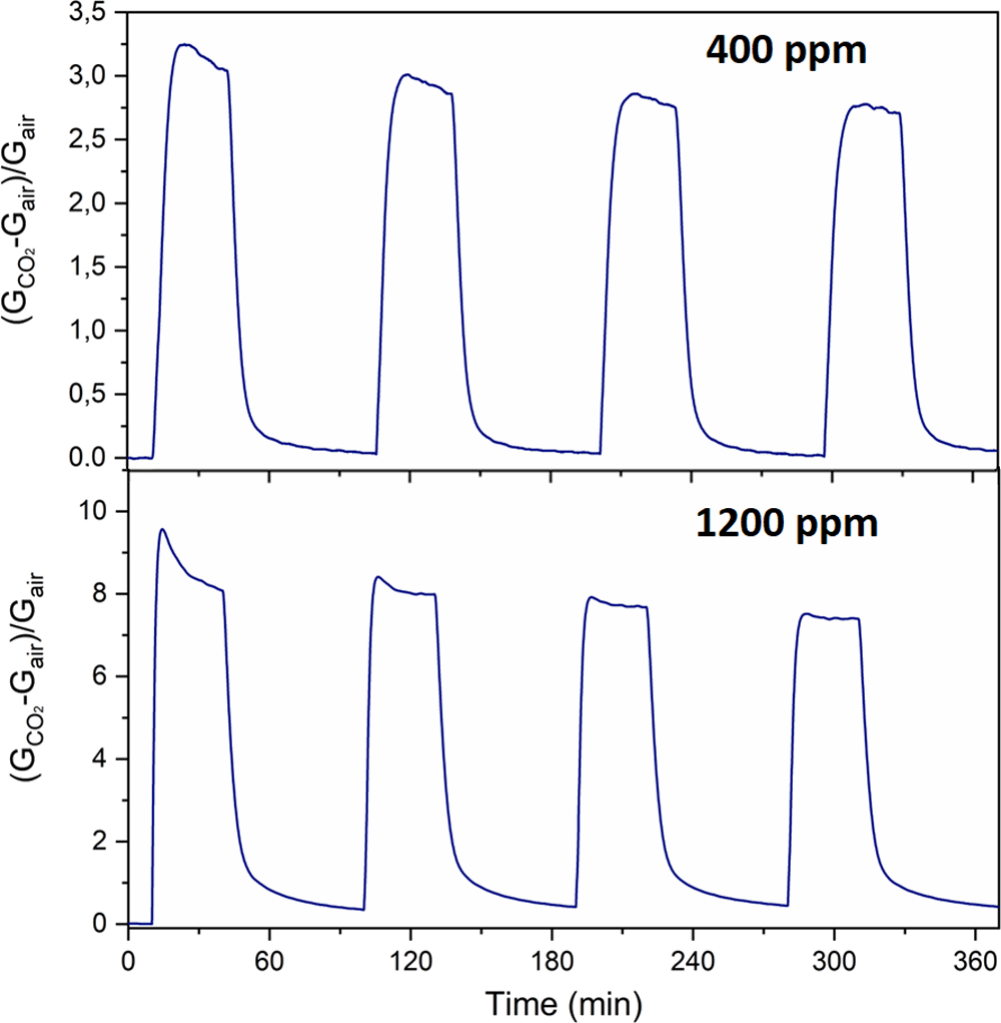
Four-cycle repeatability test as a function of time. For these experiments, the Na:In_2_O_3_ sensor was exposed toward 400 and 1200 ppm of CO_2_ gas. During the measurements, the temperature and humidity inside the chamber were 25 °C and 2 RH%, respectively.

The film response also proved to be repeatable (see Figure 5), as requested for the application as a sensor. The response τ_res_ and recovery τ_rec_ times turned out to be 5 and 9 min at 400 ppm and 3 and 23 min at 1200 ppm. The kinetics of the reaction mechanism could be accelerated by increasing the working temperature; however, already at this level, the sensors are adequately prompt to address indoor and outdoor applications.

Response and recovery times of the order of 10 min are typical characteristics timing for other MOX semiconductors as in previous works.^58−60^

Under dry conditions, n-type semiconductors form an electron depletion region near the surface due to adsorption, dissociation, and ionization of environmental oxygen in the operating temperature range of 200–500 °C, which leads to decreasing the sensor conductance. Thus, a chemical reaction between the negatively charged surface oxygen and any reducing gaseous analyte releases electrons to the conduction band (CB), resulting in an increase of the film conductivity. Under wet conditions, the conductance of a MOX semiconductor is affected by the dissociation of water vapor on the surface forming protons and hydroxyl groups (eq 5).^40^ Protons react with chemisorbed O^–^ species forming neutral −OH groups, thus affecting the conductance.
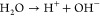
5On the other hand, OH^–^ replaces the coverage of O^–^ species, passivating the film adsorption sites for detection of analytes. Moreover, in a humid environment, the gas sensing response of a MOX semiconductor is attributable to the competition between the adsorption/desorption processes of H_2_O molecules and the analyte.

**Figure 6:**
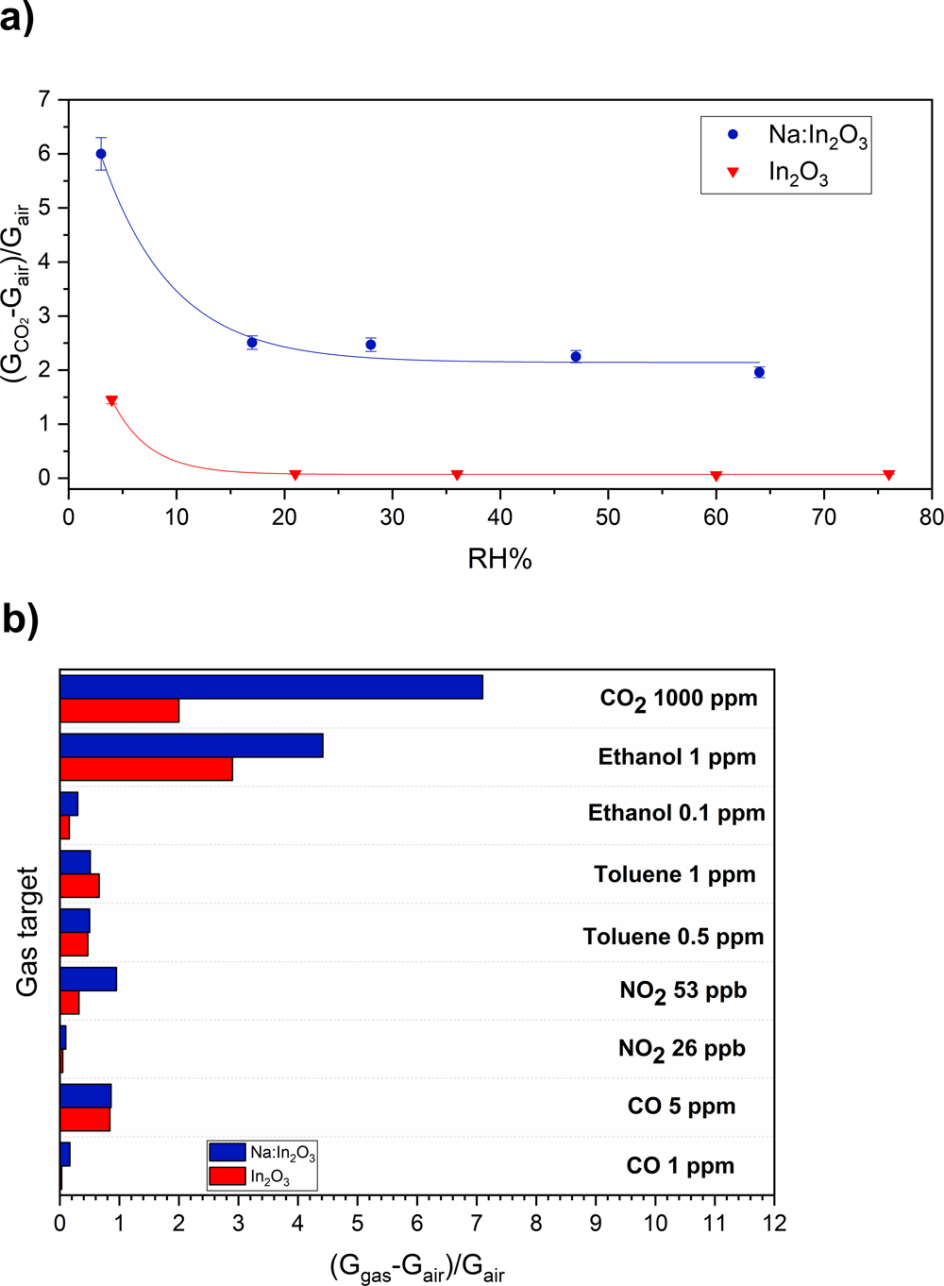
(a) Influence of RH% on the response to 500 ppm of CO_2_ for Na:In_2_O_3_ and pristine In_2_O_3_ sensors. (b) Selectivity of In_2_O_3_-based films toward different analytes. During the measurements, the temperature and humidity inside the chamber were 25 °C and 2 RH% (except for wet analyses), respectively.

In this study, the Na:In_2_O_3_ sensor was exposed to 500 ppm of CO_2_ at different RH levels (3–64%). As shown in Figure S8 (Supporting Information), with an increase of relative humidity, the baseline of film conductance also increased due to the formation of the −OH groups. With the injection of CO_2_, competition for the active sites may occur between water vapor and the target gas. Hence, the combination of such competitive interactions resulted in a substantial change in film conductance. Indeed, in the presence of humidity, the sensor exhibited a lower response (eq 2) to CO_2_ than in dry conditions (Figure 6a). It is worthwhile noticing that the response is marginally affected by humidity over a wide range, which is a key feature for possible applications. By contrast, Figure 6a shows that the undoped In_2_O_3_ film response merely vanishes under wet conditions.

Moreover, in the practical implementation of a sensing unit vs an analyte of interest, one should consider the co-presence of other gases whose physicochemical properties may affect the sensor response. Therefore, Na:In_2_O_3_ film selectivity was explored by exposing the sensors to various interferents, such as ethanol, toluene, NO_2_, and CO at different concentrations (Figure 6b). These were selected in sight of indoor and outdoor air quality monitoring, according to the concentration of interest reported by NIOSH and ASHRAE.^36−39^

It turned out that the Na-doped sensor showed better responses than the pristine In_2_O_3_ sensor for all the gases tested with a theoretical LOD of ∼4.1 ppb.^41^ In particular, we estimated the *k*_s_(40) of the sensors for CO_2_ detection (Table S3, Supporting Information). Hence, the results obtained make the achieved sensor attractive for various CO_2_ applications, since it is not significantly affected by the influence of other interfering gases.

To further evaluate the features of the Na:In_2_O_3_ sensor proposed in this work, we compare its sensing performance vs CO_2_ to those of other MOX-based sensors, which have been reported in the literature so far (Table 2). The table below summarizes the investigations conducted on CO_2_-sensitive materials operating at relatively low temperatures and prepared by synthesis methods similar to the one used in this work.

**Table 2:** Sensing Parameters of Different CO_2_ Sensors Based on MOX Semiconductors

sensing material	synthesis route	operating temperature [°C]	response at concentration [ppm]	operating condition	reference
ZnO/CuO nanorods	hydrothermal	RT	0.09 at 1000^a^	dry	(16)
5wt%Sn-CdO nanopowders	co-precipitating	250	1.18 at 5000^b^*	dry	(17)
CuO@1wt%Ag-BaTiO_3_ spheres decorated	mixing	120	0.60 at 1000^a^*	dry	(18)
CeO_2_ yolk-shell nanospheres	solvothermal	100	3.98 at 2400^c^	negligible response below 70 RH%	(19)
Nd_2_O_2_CO_3_ nanoparticles	sol–gel	350	4.00 at 1000^d^	negligible response below 50 RH%	(20)
CaO-In_2_O_3_ mesoporous	impregnation	230	1.80 at 2000^d^	dry	(21)
N-ZnO nanoparticles	sol–gel	250	4.50 at 2000^d^*	dry	(61)
Na:In_2_O_3_ nanoparticles	sol–gel	200	7.10 at 1000^e^	uniform response above 15 RH%	this work

a , where *R* is the resistance (*R*_gas_, resistance in CO_2_ atmosphere; *R*_air_, resistance in carrier gas). The asterisk denotes a value not explicitly stated in the study but approximated from a graphical plot.

b , where *R* is the resistance (*R*_gas_, resistance in CO_2_ atmosphere; *R*_air_, resistance in carrier gas).

c , where *R* is the resistance (*R*_gas_, resistance in CO_2_ atmosphere; *R*_air_, resistance in carrier gas).

d , where *R* is the resistance (*R*_gas_, resistance in CO_2_ atmosphere; *R*_air_, resistance in carrier gas). The asterisk denotes a value not explicitly stated in the study but approximated from a graphical plot.

e , where *G* is the conductance (*G*_gas_, conductance in CO_2_ atmosphere; *G*_air_, conductance in carrier gas).

It is noteworthy that the sensor in this work exhibits superior functionality vs CO_2_. First, with respect to sensing devices produced by the same synthesis route, it operates at lower temperatures. Further, above all, its sensitivity ranks among the best and it is marginally affected by environmental humidity, especially it is indifferent over a very wide range (15–64 RH%), paving the way to indoor and outdoor applications.

### *Operando* DRIFT Investigation toward CO_2_ Detection

To elucidate the sensing mechanism that enables the electrical activity of the new material in the presence of CO_2_, *operando* DRIFT measurements were performed. This advanced characterization technique provides an insight into the species adsorbed at the surface, aiding the analysis of the products formed as a result of chemical reactions.

The behavior of Na:In_2_O_3_ was first compared to that of the pristine In_2_O_3_. Each sensor was exposed to synthetic dry air, to identify the species pre-adsorbed onto the material surface before supplying CO_2_. The single-channel spectra were collected, while each sensor was being heated at the same working temperature of 200 °C to compare their sensing mechanism in the same thermodynamics conditions (Figure S9, Supporting Information). Simultaneously, the resistance of the sensing films was acquired.

Second, the sensors were exposed to 3500 ppm of CO_2_ in dry conditions (Figure S10a, Supporting Information). Comparing the two sensors, there is the formation of bridged carbonates, bidentate for Na:In_2_O_3_ and inorganic carboxylates for In_2_O_3_. According to the literature,^62^ the latter, due to their thermal stability, are less reactive with respect to the former species, and this is explanatory of the limited electrical response of pristine In_2_O_3_ vs CO_2_.

**Figure 7:**
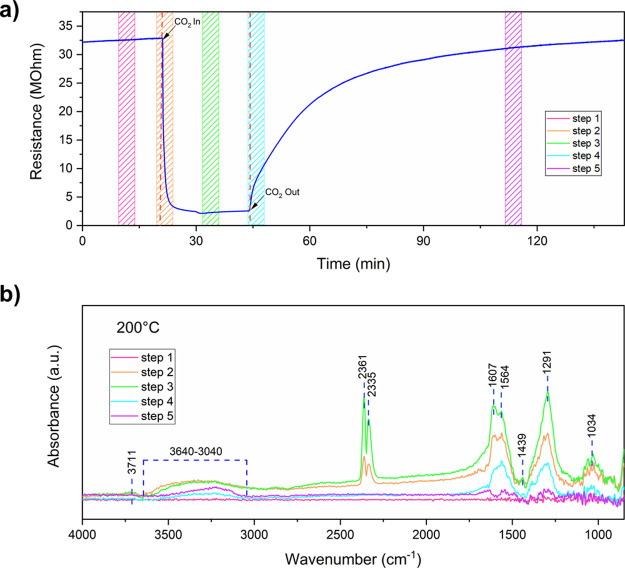
(a) Measurement scheme, resistance change when the gas sensor was exposed to 1000 ppm of CO_2_ in dry air at 200 °C with time intervals marked for FTIR spectrum sampling. (b) Spectra acquired during CO_2_ input and output under dry air conditions at time intervals marked above.

The solid–gas kinetics occurring at the surface of the Na:In_2_O_3_ sensor was investigated by acquiring the DRIFT spectra at topic intervals, while the dynamical response of the sensor vs 1000 ppm CO_2_ was being carried out (Figure 7). The figure highlights that adsorption and desorption are completely reversible at 200 °C. The DRIFT spectra highlighted the presence of hydroxyl groups adsorbed on the surface during CO_2_ injection (see steps 2 and 3; Figure 7). According to DRIFT studies using D_2_O/H_2_O on In_2_O_3_ by Boehme et al.,^63^ the sharp peak at ca. 3711 cm^–1^ can be attributed to the formation of isolated hydroxyl groups, while the broad bands between 3640 and 3040 cm^–1^ are bridged hydroxyls. Both species are due to residual water vapor in the gas injection tube (see Figure S11a, Supporting Information), and they decreased during the recovery to dry conditions (see step 4; Figure 7). However, gaseous CO_2_ molecules were the most abundant species observed by DRIFT.^64^ As shown in Figure 7b, the presence of the characteristic peaks at ca. 2339 and 2365 cm^–1^ belongs to these molecules (see steps 2 and 3; Figure 7). The bands at 1439 and 1034 cm^–1^ are assigned to In-O lattice vibration overtones.^65^

In the region between 1600 and 1000 cm^–1^, there are two main carbonate species. Indeed, the exposure of the sample to CO_2_ flow led to the rapid formation of bidentate carbonate (b-HCO_3_^–^) as recorded by 1607 and 1291 cm^–1^, while bridged bidentate carbonate (br-CO_3_^2–^) was at 1564 cm^–1^.^66^

From the electrical point of view, the sensor resistance decreased with the injection of CO_2_ due to carbonate species and hydroxyl group formation (steps 2 and 3; Figure 7a). On the other hand, film resistance increased as a result of CO_2_ desorption (steps 4 and 5; Figure 7a), which led to a significant decrease in carbonate species, while the bands corresponding to −OH groups remained almost constant in intensity owing to the lower working temperature (200 °C), which inhibited their desorption.

To further investigate the sensing mechanism toward CO_2_, we compared the DRIFT spectra acquired during the exposure to the two different concentrations of CO_2_ (1000 and 3500 ppm) (Figure S10b, Supporting Information). At higher concentrations, a slight increase in the intensities of all adsorbed species was recorded and two more peaks showed up at 3627 and 3600 cm^–1^. According to the literature,^66^ they can be attributed to the ν(OH) modes of the newly formed hydroxyl groups of the bidentate carbonates and monodentate carbonates.

**Figure 8:**
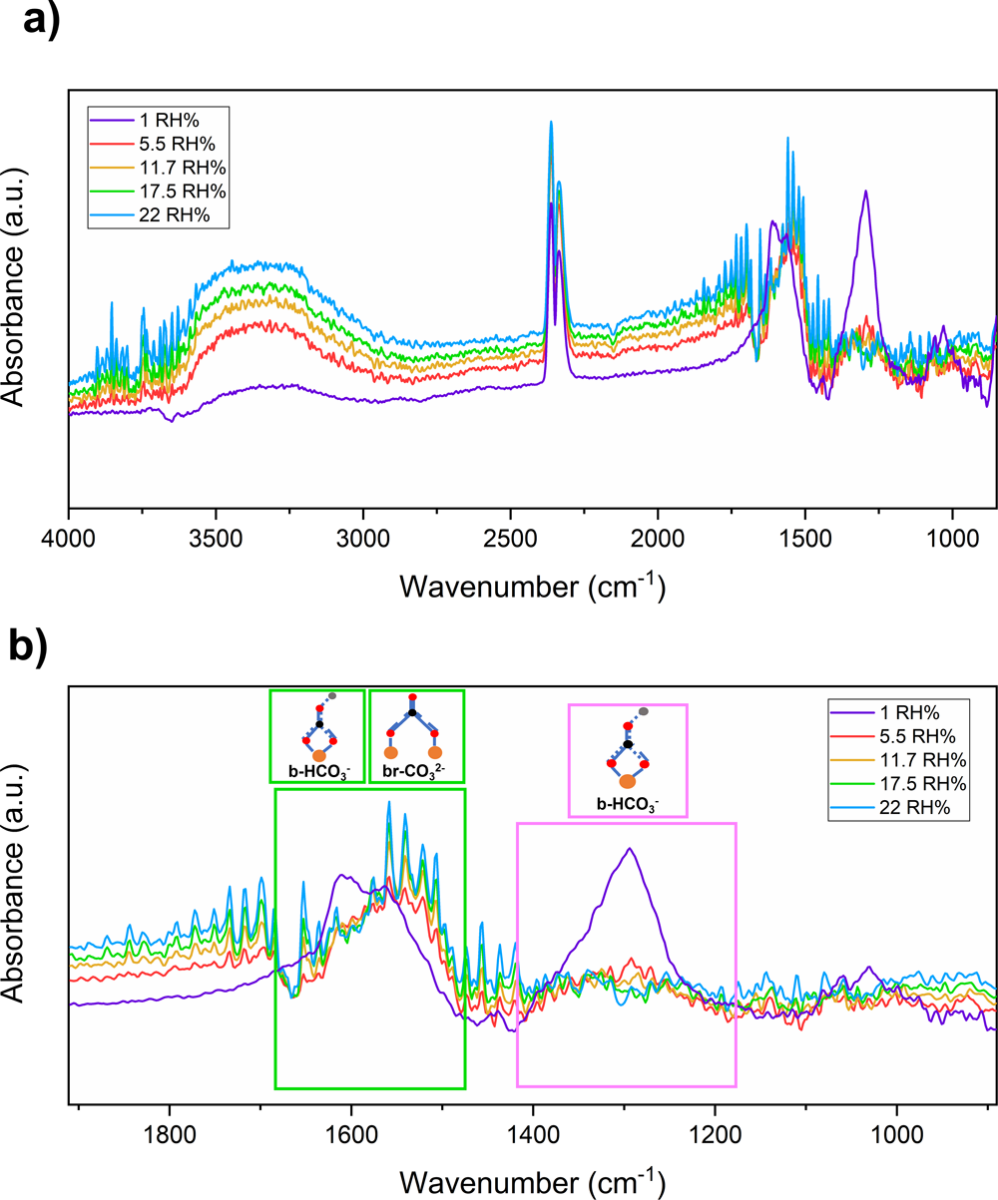
(a) Spectra of the Na:In_2_O_3_ film at 200 °C acquired during the inlet of 1000 ppm of CO_2_ in dry conditions (1 RH%) and in wet conditions. (b) Magnification of the spectrum reported in panel (a) in the range from 2000 to 850 cm^–1^. The spectral noise is related to the increase of water species adsorbed at the surface.

The stimulating behavior under humidity exposure we observed led us to increase the relative humidity concentrations (5.5–22 RH%). Under wet environments in the presence of 1000 ppm of CO_2_ (see Figure 8), the DRIFT spectra can be explained in terms of chemical activity of three types of adsorbates on the surface, namely, −CO_3_^2–^, −OH^–^, and −O^–^ (see Figure 8a). The narrow and intense peaks between 4000–3300 and 2100–1300 cm^–1^, which increased with the humidity concentrations, are assigned to the gaseous H_2_O molecules.^67^ The competitive relation between hydroxyl groups and carbonate species became clear as an increase in the peaks due to hydroxyl groups resulted in a decrease of the carbonate species (see Figure 8b). Therefore, adsorbed H_2_O on the surface limits the adsorption sites and inhibits the formation of carbonates.^64^

### Sensing Mechanism

**Figure 9:**
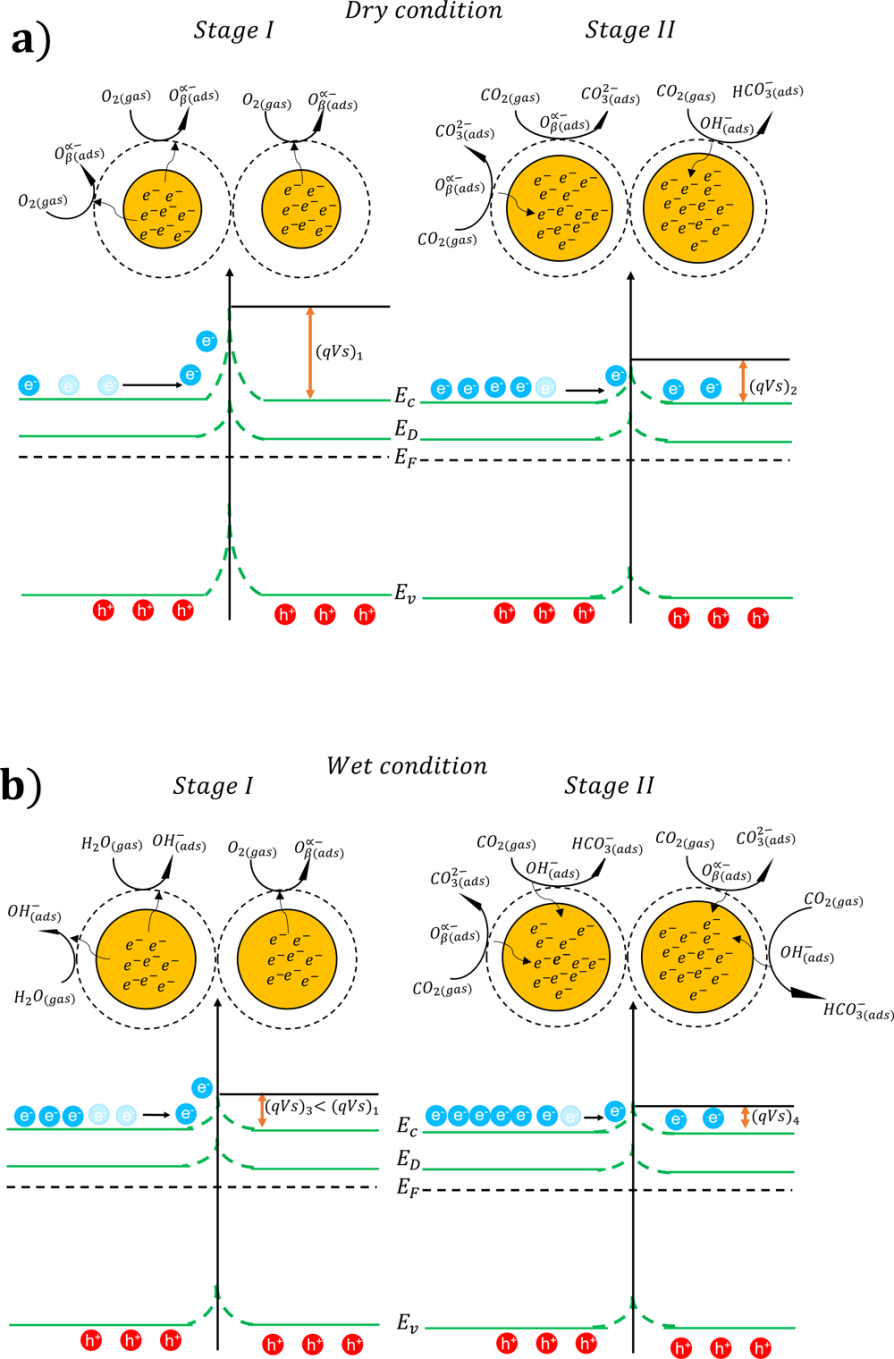
Reaction mechanisms on the surface (a) in dry air (stage I) and under CO_2_ exposure (stage II) and (b) in wet air (stage I) and under CO_2_ exposure (stage II). *E*_c_ and *E*_v_ are the conduction and valence bands, *E*_F_ is the Fermi level, and *E*_D_ is the donor level created due to the doping by Na.

By combining the information obtained through DRIFT measurements with the electrical characterization of the film, it was possible to formulate some hypotheses about the detection mechanism of CO_2_ by Na:In_2_O_3_ as a chemoresistive functional material. In particular, the sensing mechanism can be explained in terms of the band bending theory.^68,69^ Considering dry air conditions, at working temperatures ranging between 100 and 500 °C, the interaction of the sensing layer with atmospheric oxygen leads to ionosorption of the latter in molecular (O_2_^–^) and/or atomic (O^–^, O^2–^) species (see eqs 6–9).^70^ More precisely, the reactions occurring at the surface, listed as a function of increasing temperature, are the following:
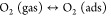
6
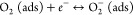
7
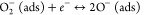
8
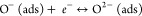
9At an operating temperature of 200 °C, the oxygen ions (O^–^) would dominate the ionosorption (eq 8).^71^ As a result, atmospheric oxygen traps electrons from the CB, creating a potential barrier (q*V*_s_)_1_ and consequently increasing the resistance of the film with respect to room-temperature operation (stage I in Figure 9a). As CO_2_ is fed (stage II in Figure 9a), its molecules react with the pre-adsorbed oxygen ions at the surface following eq 10 to produce carbonate ions and electrons.^1,72^
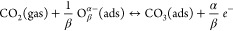
10Therefore, the concentration of free electrons in the CB of Na:In_2_O_3_ increases, leading to a decrease in the potential barrier in this new state, (q*V*_s_)_2_, and consequently in the film resistance.

Under wet conditions, water molecules (eq 5) both react with and hinder chemisorbed oxygens at the surface (stage I in Figure 9b). This phenomenon influences the band bending effect, i.e., the potential barrier created in wet air is lower than in dry air ((q*V*_s_)_3_ < (q*V*_s_)_1_). This results in a decrease of the film resistance. Exposing the sensor to CO_2_ in wet conditions, hydroxyl groups promote the reaction in eq 11, decreasing the potential barrier ((q*V*_s_)_4_ < (q*V*_s_)_3_) (stage II in Figure 9b).
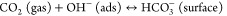
11However, a higher water concentration hinders the reaction between active sites with CO_2_. Therefore, the variation of potential barrier height with the exposure to the analyte is lower in wet than in dry conditions, inducing a lower response.

The role of sodium in enhancing the CO_2_ detection relies on the increase in the concentration of oxygen vacancies as confirmed by XPS analysis (see Table 2 and Figure 2), according to the following reaction:^73^
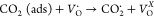
12where *V* _O_^·^ denotes the singly ionized oxygen vacancy and *V*_O_^*X*^ denotes the neutral oxygen vacancy.

On the one hand, whereas oxygen vacancies are additional surface sites of high reactivity, sodium could itself prove to be a promoter of the catalytic effect,^74^ causing both electronic and redox changes in the material.

## Conclusions

In this work, Na:In_2_O_3_ nanostructured powder was synthesized by the sol–gel method. Extensive characterization by SEM, TEM, XRD, XPS, and UV–vis confirmed the nanostructured nature of the material as spherical particles and the presence of sodium dispersed in the crystalline structure. Indeed, aggregates or second phases were not observed by TEM and XRD analysis, suggesting that the addition of sodium is properly distributed. In particular, XPS revealed an increase in the concentration of oxygen vacancies in the doped sample as compared to the pristine one, which turned out to magnify the surface reactivity of the doped material. Indeed, Na:In_2_O_3_ was capable of reducing CO_2_, suggesting that it can straight be used as a gas sensor.

The sensing performance was evaluated by investigating the sensitivity in dry and in RH% conditions, repeatability, and selectivity. In particular, the negligible influence of humidity enables the new MOX-based sensing material to a wide scenario of possible applications.

By comparing the performance to other CO_2_ sensors in the literature, it emerged that Na:In_2_O_3_ as a functional material is very effective for CO_2_ sensing at low temperatures. To achieve additional information on the surface reaction mechanism of Na:In_2_O_3_-based gas sensors to CO_2_, we performed *operando* DRIFT spectroscopy measurements. It was highlighted that the adsorption of CO_2_ on the surface led to the formation of carbonate species, which, in the case of wet conditions, compete with hydroxyl groups on the film. To further investigate this process, it might be necessary to conduct H_2_O/D_2_O and ^12^CO/^13^CO isotope exchange experiments to discriminate the different groups on the surface.

Therefore, the enhancement of oxygen vacancies caused by sodium doping was fundamental to increase the reactivity vs CO_2_. To deeply explore the role of sodium in the sensing mechanism, density functional theory calculations might provide significant information on the electronic structure and energy of interactions.

These sensing capabilities, corroborated by the technological advantage to operate at low temperatures and the possible miniaturization for IoT networks, make the material investigated in this work the most effective MOX-based gas sensor for CO_2_ detection, competing with widespread optical sensors.

## Supporting Information Available

Sensor fabrication method, *operando* drift setup, material characterization, electrical characterization, and *operando* drift investigation toward CO_2_ detection (PDF)
