# Supplementary material for: Functionalization of Indium Oxide for Empowered Detection of CO2 over an Extra-Wide Range of Concentrations
Source: ACS Appl Mater Interfaces. 2023 Jun 30;15(28):33732–43. doi: 10.1021/acsami.3c04789 (PMC10360036; doi:10.1021/acsami.3c04789)
Supplement: Supplementary file 1 — am3c04789_si_001.pdf [file am3c04789_si_001.pdf]

Supporting Information

Functionalization of Indium Oxide for Empowered Detection of CO_2 over an Extra-Wide Range of Concentrations

A. Rossi[1], B. Fabbri[1], E. Spagnoli[1], A. Gaiardo[2], M. Valt[2], M. Ferroni[3, 4], M. Ardit[1], S. Krik[5], A. Pedrielli[2], L. Vanzetti[2], V. Guidi[1]*

[1] Department of Physics and Earth Sciences, University of Ferrara, Via Saragat 1, Ferrara 44122, Italy

[2] MNF- Micro Nano Facility, Sensors and Devices center, Bruno Kessler Foundation, Via Sommarive 18, Trento 38123, Italy

[3] Institute for Microelectronics and Microsystems IMM-CNR, via Gobetti 101, 40129 Bologna, Italy

[4] Department of Civil, Environmental, Architectural Engineering and Mathematics (DICATAM) Università degli Studi di Brescia - Via Branze, 43 - 25123 Brescia

[5] Sensing Technologies Lab, Faculty of Engineering, Free University of Bozen-Bolzano, Piazza Università 5, Bolzano 39100, Italy

* Corresponding Author e-mail: arianna.rossi@unife.it

SENSOR FABRICATION METHOD

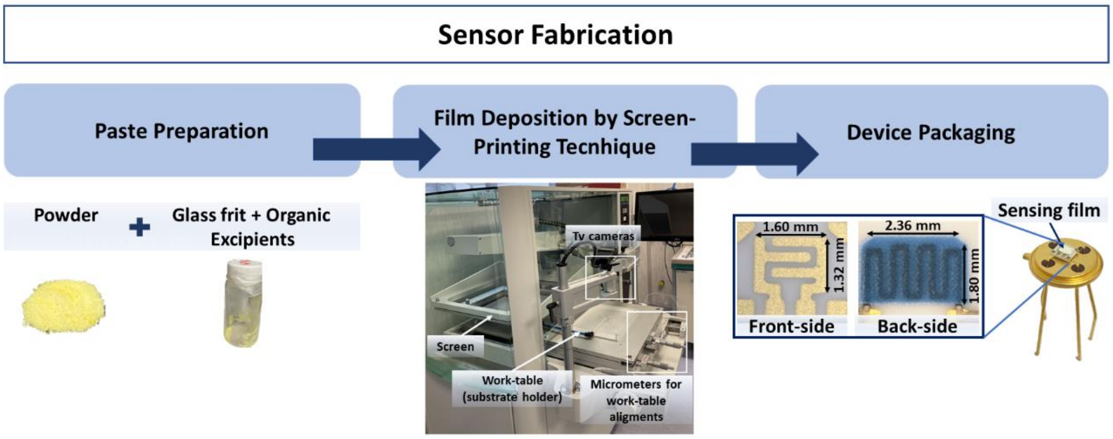

Figure S1. Schematic representation of the manufacturing process of chemoresistive gas sensors: paste preparation (step 1), film deposition (step 2), and sensor packaging (step 3).

OPERANDO DRIFT SETUP[1,2]

The system was designed to match the optics of Harrick's Praying Mantis mirror, embedded in the sample compartment of Bruker Vertex 70 V vacuum FT-IR spectrometer. The sensor test chamber, placed inside the Praying Mantis, consists of three parts: the main cell body, a cell holder, and a vacuum-compatible precision XY microstage (Standa), which is coupled to the axis already present in Harrick's chamber. Two Viton O-rings provide a seal between the two components of the cell body and the IR dome. The main chamber consists of a SS frame with two monolithic ZnSe windows for the IR beam path and a SiO window for visual alignment, made possible by a precision microstage. Connections for electrical, sensor heating, and T/RH% measurements are established via JST connector. To perform electrical measurements on the sensing film under gas flow during DRIFT analysis, a dedicated custom instrument was developed both at the hardware level, adapting all materials and wiring used for vacuum operation, and at the software level. The setup was designed to be able to perform a complete electrical characterization of the sensor, with precise control of the working temperature and monitoring of the temperature and RH% inside the test chamber using a commercially available

Sensirion SHT3X sensor. This circuit can set a voltage drop within - 12 and +12 V and measure positive and negative current from 8 pA to 167 mA. Precise temperature control is achieved by a proportional-integral-derivative (PID) controller. Temperature control of the sensing film, with a resolution of 1 °C, is achieved by supplying a precise amount of current to the previously calibrated platinum heater. The hardware is controlled by the user via a graphical user interface (GUI) developed in Java, which allows the user to set all measurement parameters and monitor the collected data in real time. The gas compound delivery system, on the other hand, is equipped with four calibrated mass flow controllers (MFCs) (Brooks, SLA5800) and a secondary electronic control module (Brooks, 0260). Humidity control within the chamber is achieved by a dedicated MFC that flows certified synthetic air through a bubbler filled with deionized water.

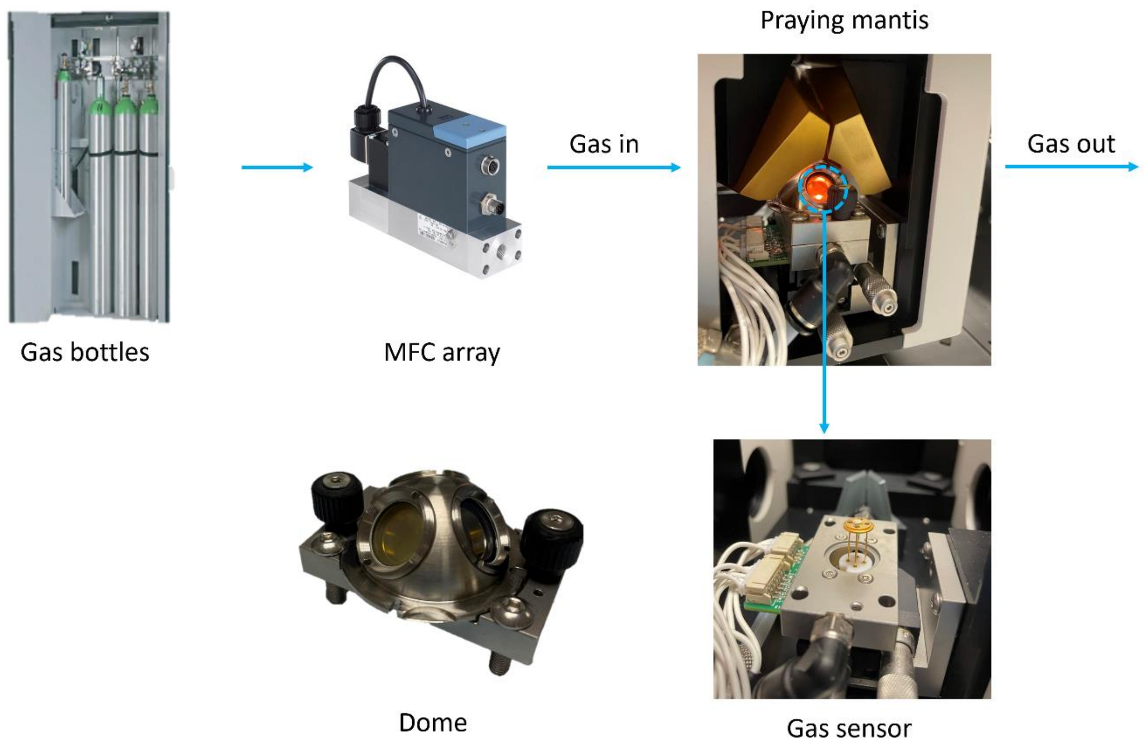

Figure S2. Schematic representation of *operando* DRIFT experimental setup.

MATERIAL CHARACTERIZATION

X-ray diffraction. XRD data collection was performed on a Bruker D8 Advance Da Vinci diffractometer working in Bragg-Brentano geometry, and equipped with a Cu-anode X-ray tube, Ni-filter to suppress the Cu $K\beta$ component, and a LynxEye XE silicon strip detector (angular range covered by the detector = 2.585° 2θ) set to discriminate Cu $K\alpha_{1,2}$ radiation. The powder was loaded in a 2 mm-deep cavity in a poly (methyl methacrylate) specimen holder and scanned in a continuous mode from 5 to 90° 2θ, with step size of 0.02° 2θ and a counting time of 2 s per step. Qualitative phase analysis of collected patterns was performed by means of the Bruker AXS EVA software (v.6.0.0.7). Collected XRPD patterns were modeled by means of the fundamental-parameter Rietveld approach (TOPAS v.5.0, Bruker).

Scanning electron microscopy. The morphology of the obtained material was investigated by SEM using a Zeiss LEO 1530 FEG microscope, equipped with an Oxford Inst. INCA 250 30 mm^2 SSD EDX spectrometer.

a)

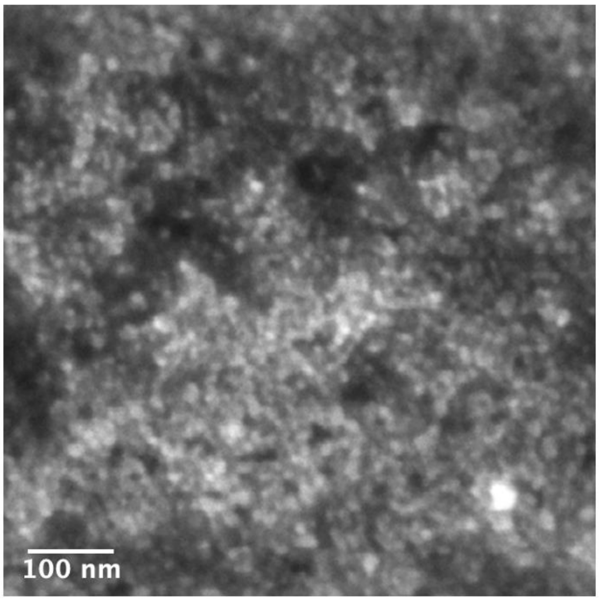

b)

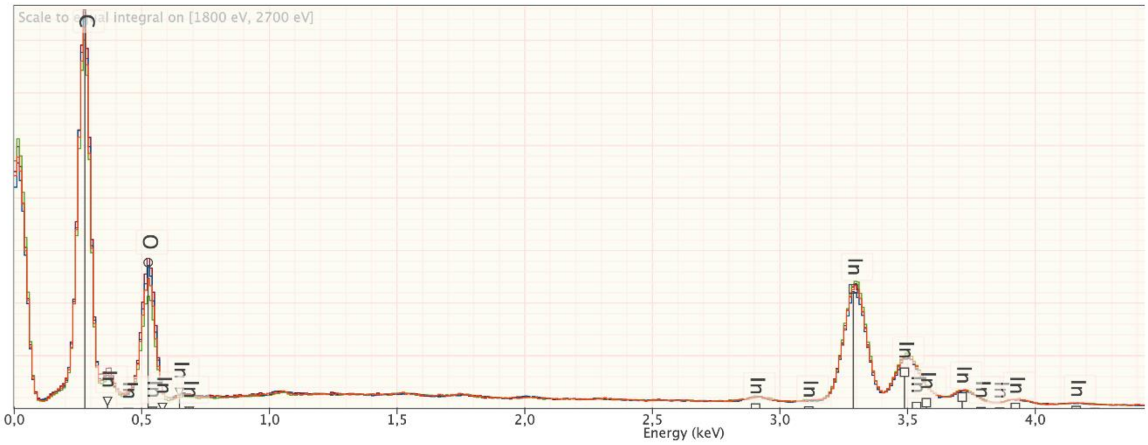

Figure S3. a) SEM image and b) relative EDX of In_2O_3 powder.

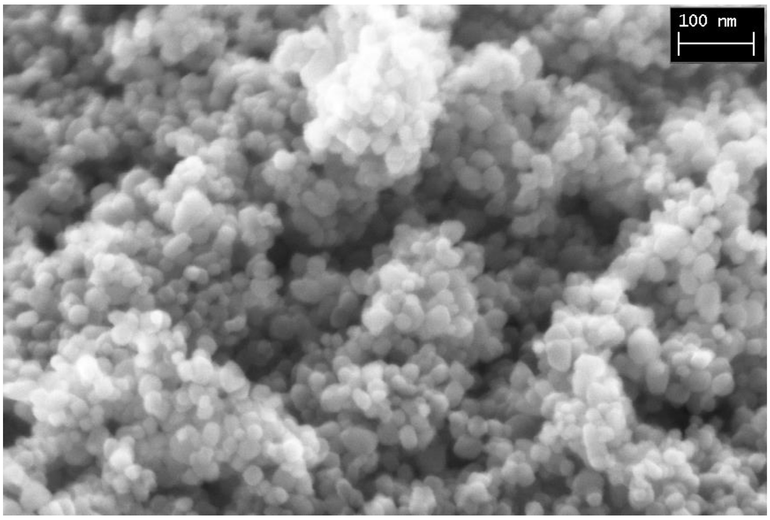

Figure S4. High-magnification SEM image of $Na:In_2O_3$ powder.

Transmission electron microscopy. Microstructural and compositional analyses were performed by using a Philips TECNAI F20 ST TEM operating at 200 kV. The instrument was equipped with a EDAX SUTW EDX spectrometer and Fischione Inst. High-Angle Annular Dark-Field STEM imaging detector. TEM images were acquired in phase contrast mode. The sample was ground and suspended in iso-propanol solution and sonicated. Then a few drops of the solution were drop casting over a molybdenum grid and dried on a heating plate.

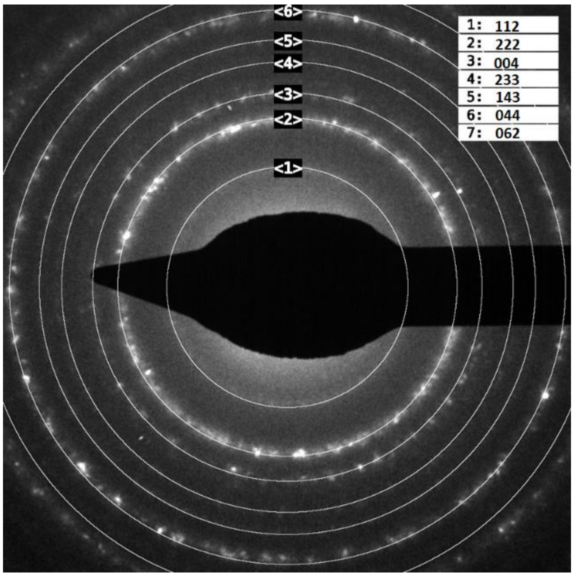

Figure S5. Selected area diffraction pattern of Na:In_2O_3 powder.

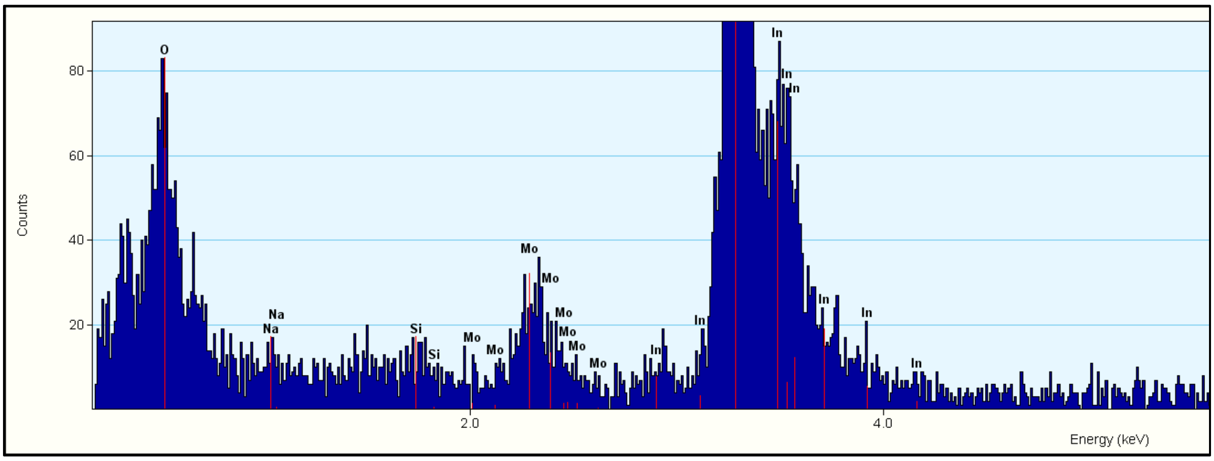

Figure S6. EDX spectrum of a Na:In_2O_3 nanoparticle from STEM analysis.

X-ray photoelectron spectroscopy. XPS measurements were performed using a Kratos AXIS Ultra^{DLD} instrument (Kratos Analytical, Manchester, UK) equipped with a hemispherical analyzer and a monochromatic X-ray source Al Kα (1486.6 eV), in spectroscopy mode. For the measurements, powders were deposited on carbon tape, placed on a silicon support. Samples were analyzed using a 0° take-off angle between the sample surface normal and the analyzer axis, corresponding to a sampling depth of approximately 10 nm. The surveys were recorded to identify the elements present on the surface. High-resolution spectra of the core

levels of In, O and Na were collected for each sample. For the fit of the O 1s peaks we used a weighted sum of a Lorentzian and a Gaussian with a ratio of 0.25. XPS quantification was performed using the instrument sensitivity factors and high-resolution spectra. The alignment was performed by setting the hydrocarbon peak in the C 1s core level peak at 285 eV. All XPS data were analyzed using the software described elsewhere.[3]

Table S1. Elemental composition [at%] and In 3d binding energies of the In_2O_3 and $Na:In_2O_3$ powders, detected with XPS.

Sample	In $3d_{3/2}$ [eV]	In $3d_{5/2}$ [eV]	In [% at]	Na [% at]	O [% at]	C [% at]
In_2O_3	451.6	444.1	38.7	0.0	51.1	10.2
$Na:In_2O_3$	451.4	443.8	33.0	6.7	48.5	11.8

Table S2. Binding energies and relative quantitative analysis of the deconvolution O 1s peaks for In_2O_3 and $Na:In_2O_3$.

Sample	O lattice (In-O-In)		Oxygen vacancies		OH-In		H_2O	
	Binding Energy [eV]	%peaks vs. O_{tot} [area]	Binding Energy [eV]	%peaks vs. O_{tot} [area]	Binding Energy [eV]	%peaks vs. O_{tot} [area]	Binding Energy [eV]	%peaks vs. O_{tot} [area]
In_2O_3	529.6	65.0	530.3	7.0	531.4	27.9	533.1	0.1
$Na:In_2O_3$	529.2	58.3	529.9	8.1	531.1	32.0	533.0	1.6

Optical absorption analysis. UV-visible measurements were performed by using a JASCO V-670 dual beam spectrophotometer. The instrument is equipped with a deuterium lamp (190-350 nm) and a halogen lamp (330-2700 nm). The measurements were performed in the wavelength range 200-800 nm, with a sampling interval of 1 nm. In order to carry out the analysis, the powder was dispersed in 2-propanol and subjected to an ultrasonic treatment for 30 minutes. The band gap of the powders was calculated using the Tauc Plot method, i.e., by calculating and adapting the absorption data of the nanopowders with respect to the direct transition energy:

$$\alpha h\nu = A\,(h\nu - E_g)^{\frac{1}{2}} \tag{1}$$

where α is the optical absorption coefficient, $h\nu$ is the energy of the photon, E_g is the direct band gap and A is a constant.[4] Plotting the graph of $(\alpha h\nu)^2$ as a function of the photon energy and extrapolating the linear portion of the curve up to zero absorption, the values of the E_g of the investigated materials were obtained.

ELECTRICAL CHARACTERIZATION

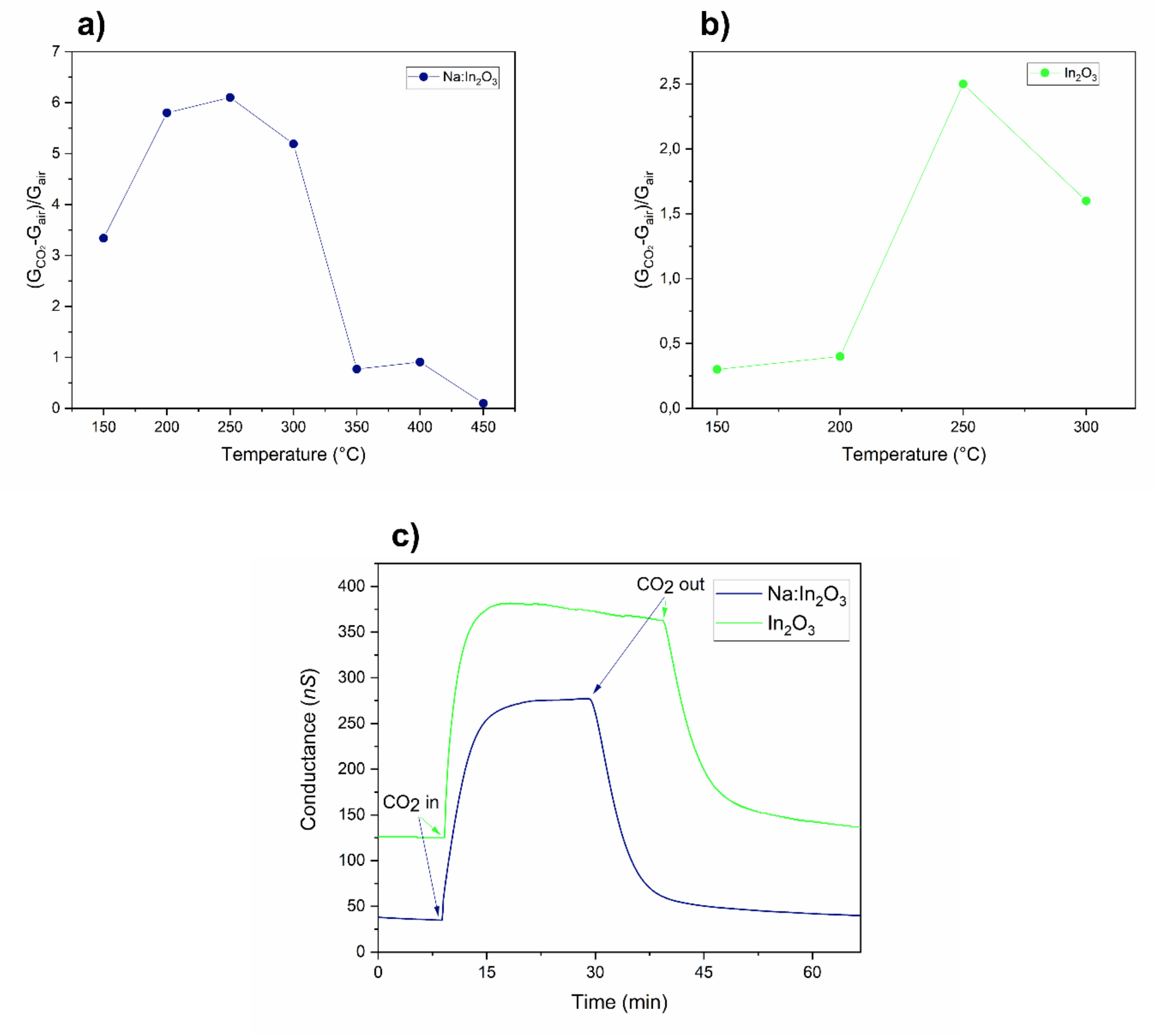

Figure S7. a) Response to 500 ppm of carbon dioxide vs. temperature for Na:In_2O_3 based sensor; b) response to 1000 ppm of carbon dioxide vs. temperature for pristine In_2O_3 . c) Conductance change of Na:In_2O_3 and pristine In_2O_3 based sensors at 200 °C and 250 °C, respectively, in presence of 1000 ppm of CO_2.

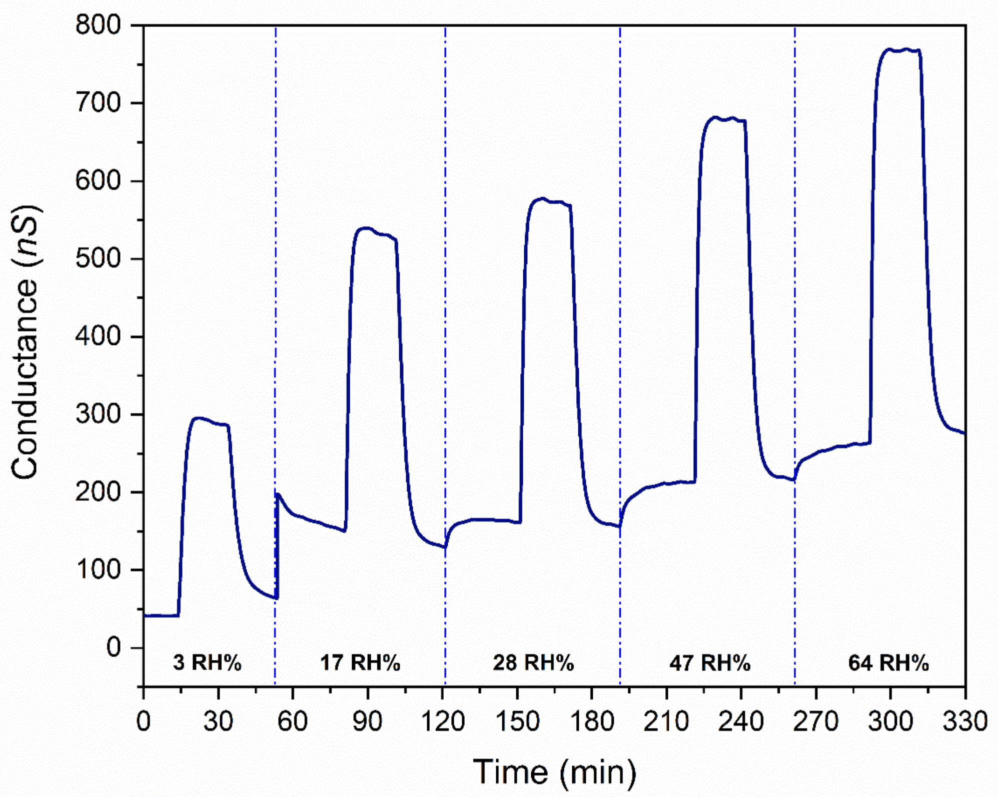

Figure S8. Conductance variation for Na:In_2O_3 based sensor exposed to 500 ppm of carbon dioxide at different relative humidity values.

Table S3. Selectivity of Na:In_2O_3 and In_2O_3 sensors at 200 and 250 °C, respectively, vs. interfering gases. The selectivity coefficient k_s was calculated using the response values of sensors towards 1000 ppm of CO_2 with respect to response values towards ethanol (0.1 ppm), toluene (0.5 ppm), NO_2 (26 ppb) and CO (1 ppm).

Sample	k_{s1} (ethanol)	k_{s2} (toluene)	k_{s3} (NO_2)	k_{s4} (CO)
Na:In_2O_3	23.6	14.2	71	42
In_2O_3	12.5	4.2	40	67

OPERANDO DRIFT INVESTIGATION TOWARDS CO_2 DETECTION

Figure S9 shows the single-channel spectra of the Na:In_2O_3 and the pristine In_2O_3 sensors operated in dry air at 200 °C. As expected, the sample exhibits distinctive characteristics of the In_2O_3 surface. Indeed, the existence of bridged (interacting) hydroxyls can be assigned to the typical broad bands between 3600 and 3000 cm^{-1}. The existence of -OH groups on the surface

of the material is suggested by the presence of a sharp band at 3300 cm^{-1}. This is due to a defect in the chamber mirrors. The three bands between 3000 and 2800 cm^{-1} are due to hydrocarbon impurities of the optical system and deleted when calculating the absorbance spectrum. As it can be clearly seen, Na:In_2O_3 sample exhibits, in the region between 1780 and 1500 cm^{-1}, the formation of a number of distinct carbonate species. The bands between 1400 and 1000 cm^{-1} are assigned to hydroxyl deformation vibrations and In-O lattice vibrations overtones.[5] Besides, some peaks appear between 1000 and 900 cm^{-1} and they can be assigned to hydroxyl deformation vibrations.

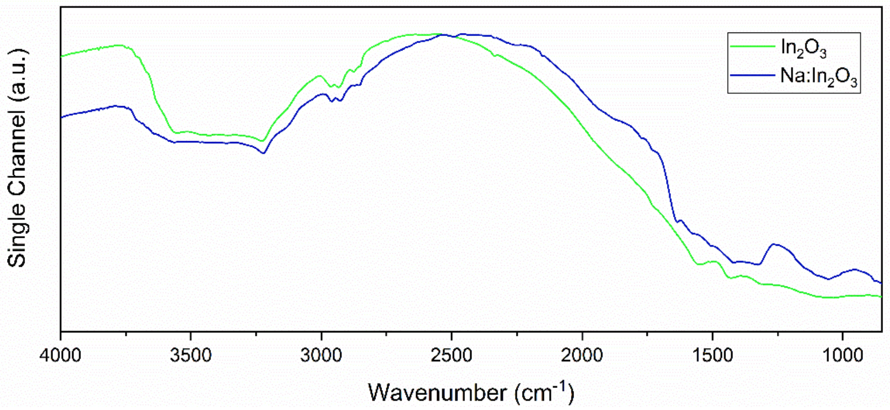

Figure S9. Single-channel spectra of Na:In_2O_3 and In_2O_3 gas sensors at 200 °C in dry air.

Figure S10a shows the absorbance spectra of the Na:In_2O_3 and the pristine In_2O_3 sensors exposed to 3500 ppm of CO_2, operated in dry air at 200 °C. We identified four peaks located between 3700 and 3600 cm^{-1} that can be assigned to -OH stretching respectively,[6] which are probably derived from residual water vapor. It can be observed for the sample Na:In_2O_3 the existence bridged (interacting) hydroxyls can be assigned to the typical broad bands between 3600 and 3000 cm^{-1}. The double band around 2365 and 2335 cm^{-1} can be assigned to gas-phase CO_2. As it can be clearly seen, Na:In_2O_3 and In_2O_3 samples exhibit, in the region between 1700 and 1300 cm^{-1}, the formation of a number of distinct carbonates species. In particular, in the case of Na:In_2O_3 there is the formation of bridged and bidentate carbonates. On the other hand,

in the case of In_2O_3, the formation of inorganic carboxylates and monodentate carbonates occurs. The bands located around 1500 and 1000 cm^{-1} are assigned to In-O lattice vibrations overtones.[5] Besides, some peaks appear between 900 and 883 cm^{-1}, and they can be assigned to hydroxyl deformation vibrations.

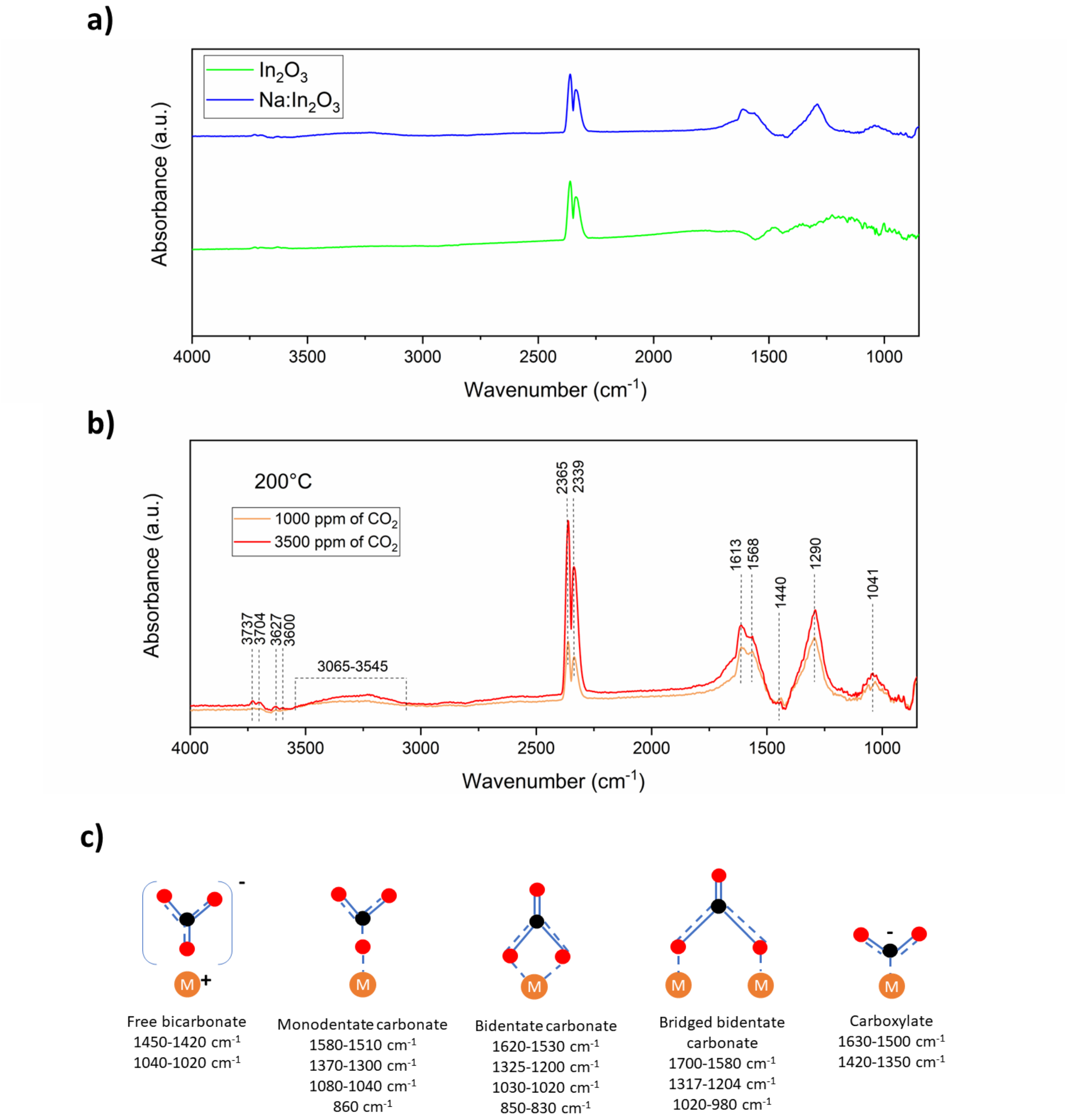

Figure S10. a) Comparison absorbance spectra of $Na:In_2O_3$ and In_2O_3 gas sensors, operating at 200 °C, exposed to 3500 ppm of CO_2 in dry air. b) Absorbance spectrum of a $Na:In_2O_3$ gas sensor at 200 °C exposed to 1000 and 3500 ppm of CO_2 in dry air. c) IR band positions (cm^{-1}) of different carbonate species on the surface.

During DRIFT measurements in dry condition, one can observe the formation of -OH groups after CO_2 injection due to a slight increase of humidity in the test chamber (1-3 RH%) (see **Figure S11a)**. These minority groups are probably due to water dissociation (**Equation 5**), which, in this case, do not effectively affect the sensing properties. In contrast, in wet condition, there is negligible "spike" formation when injecting CO_2 (**Figure S11b**).

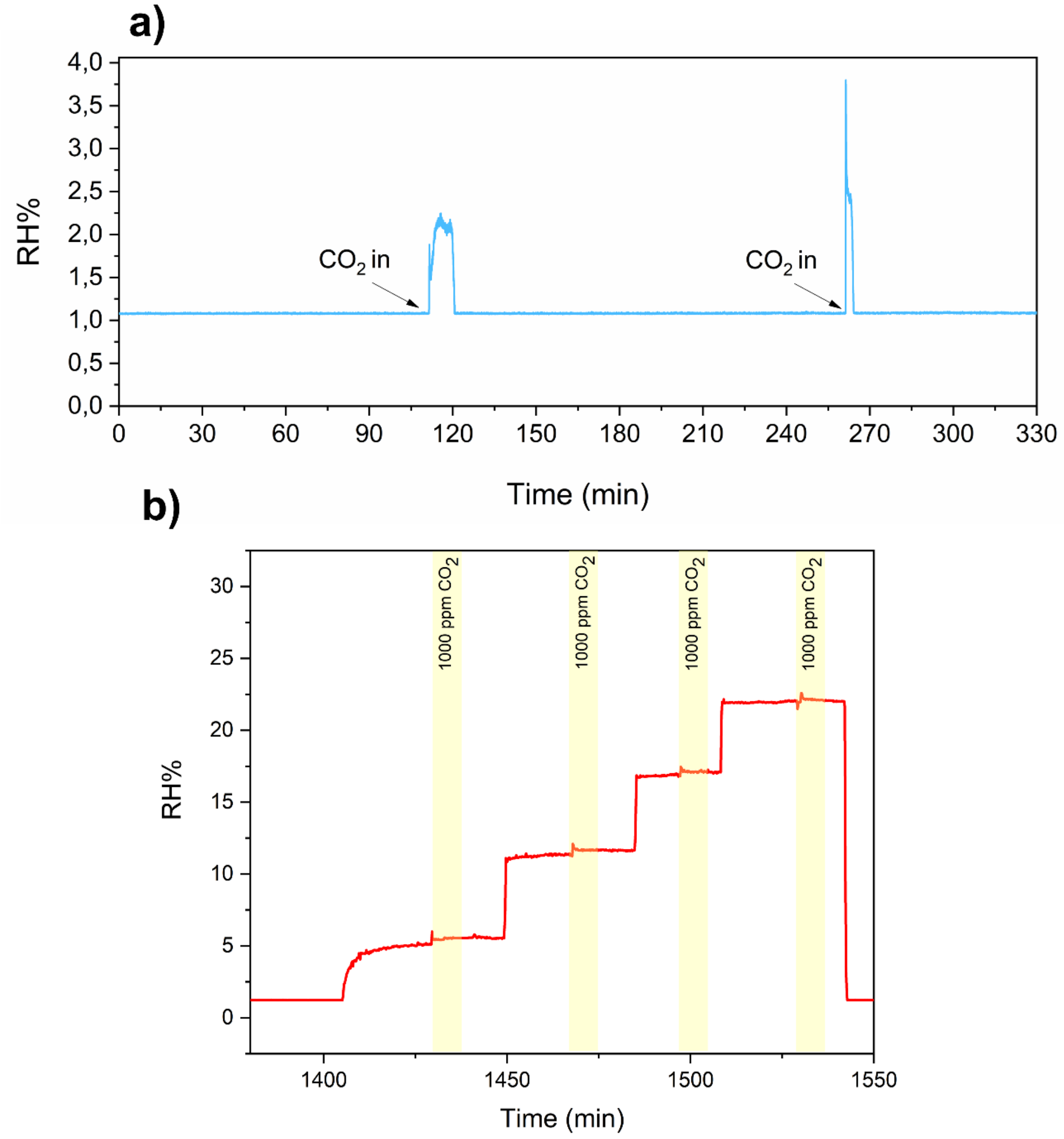

Figure S11. RH% vs. time when injecting CO_2 in the operando DRIFT test chamber a) in dry and b) wet condition.

References

(1) Valt, M.; Ciana, M. D.; Fabbri, B.; Sali, D.; Gaiardo, A.; Guidi, V. Design and Validation of a Novel Operando Spectroscopy Reaction Chamber for Chemoresistive Gas Sensors. *Sens. Actuators B Chem.* **2021**, *341*, 130012. https://doi.org/10.1016/j.snb.2021.130012.

(2) Della Ciana, M.; Valt, M.; Fabbri, B.; Bernardoni, P.; Guidi, V.; Morandi, V. Development of a Dedicated Instrumentation for Electrical and Thermal Characterization of Chemiresistive Gas Sensors. *Rev. Sci. Instrum.* **2021**, *92* (7), 074702. https://doi.org/10.1063/5.0053635.

(3) Speranza, G.; Canteri, R. RxpsG a New Open Project for Photoelectron and Electron Spectroscopy Data Processing. *SoftwareX* **2019**, *10*, 100282. https://doi.org/10.1016/j.softx.2019.100282.

(4) Ye, L.; Tian, L.; Peng, T.; Zan, L. Synthesis of Highly Symmetrical BiOI Single-Crystal Nanosheets and Their {001} Facet-Dependent Photoactivity. *J. Mater. Chem.* **2011**, *21* (33), 12479. https://doi.org/10.1039/c1jm11005e.

(5) Can, I.; Weimar, U.; Barsan, N. Operando Investigations of Differently Prepared In2O3-Gas Sensors. In *Proceedings of Eurosensors 2017, Paris, France, 3–6 September 2017*; MDPI, 2017; p 432. https://doi.org/10.3390/proceedings1040432.

(6) Grossmann, K.; Pavelko, R. G.; Barsan, N.; Weimar, U. Interplay of H2, Water Vapor and Oxygenat the Surface of SnO2 Based Gas Sensors – An Operando Investigation Utilizing Deuterated Gases. *Sens. Actuators B Chem.* **2012**, *166–167*, 787–793. https://doi.org/10.1016/j.snb.2012.03.075.
